# Transforming growth factor-beta stimulates human bone marrow-derived mesenchymal stem/stromal cell chondrogenesis more so than kartogenin

**DOI:** 10.1038/s41598-020-65283-8

**Published:** 2020-05-20

**Authors:** E. Music, T. J. Klein, W. B. Lott, M. R. Doran

**Affiliations:** 1 Queensland University of Technology (QUT), Centre for Biomedical Technologies (CBT), School of Biomedical Sciences, Faculty of Health, Institute of Health and Biomedical Innovation (IHBI), Brisbane, Australia; 2 Translational Research Institute (TRI), Brisbane, Australia; 3 Queensland University of Technology (QUT), Centre for Biomedical Technologies (CBT), School of Mechanical, Medical and Process Engineering, Faculty of Science and Engineering, Institute of Health and Biomedical Innovation, Brisbane, Australia, Institute of Health and Biomedical Innovation (IHBI), Brisbane, Australia; 4 Australian Prostate Cancer Research Centre – Queensland (APCRC-Q), Brisbane, Australia; 5 Mater Research Institute – University of Queensland (UQ), Translational Research Institute (TRI), Brisbane, Australia; 6 National Centre for the Public Awareness of Science, Australian National University (ANU), Canberra, Australia; 7 Skeletal Biology Section, National Institute of Dental and Craniofacial Research (NIDCR), National Institutes of Health (NIH), Department of Health and Human Services,, Bethesda, Maryland USA

**Keywords:** Mesenchymal stem cells, Stem-cell differentiation, Stem-cell research

## Abstract

A previous study identified kartogenin (KGN) as a potent modulator of bone marrow mesenchymal stem/stromal cell (BMSC) chondrogenesis. This initial report did not contrast KGN directly against transforming growth factor-beta 1 (TGF-β1), the most common growth factor used in chondrogenic induction medium. Herein, we directly compared the *in vitro* chondrogenic potency of TGF-β1 and KGN using a high resolution micropellet model system. Micropellets were cultured for 7–14 days in medium supplemented with TGF-β1, KGN, or both TGF-β1 + KGN. Following 14 days of induction, micropellets exposed to TGF-β1 alone or TGF-β1 + KGN in combination were larger and produced more glycosominoglycan (GAG) than KGN-only cultures. When TGF-β1 + KGN was used, GAG quantities were similar or slightly greater than the TGF-β1-only cultures, depending on the BMSC donor. BMSC micropellet cultures supplemented with KGN alone contracted in size over the culture period and produced minimal GAG. Indicators of hypertrophy were not mitigated in TGF-β1 + KGN cultures, suggesting that KGN does not obstruct BMSC hypertrophy. KGN appears to have weak chondrogenic potency in human BMSC cultures relative to TGF-β1, does not obstruct hypertrophy, and may not be a viable alternative to growth factors in cartilage tissue engineering.

## Introduction

Cartilage has a limited capacity for self-repair, and focal defects have a propensity to degrade further, resulting in osteoarthritis (OA). OA is the leading cause of pain and disability in the western world^1^. Considerable research investment is being made towards the development of strategies for cell-based cartilage repair. Bone marrow-derived mesenchymal stem/stromal cells (BMSC) are thought to be a promising cell population for use in cartilage defect repair^2^. To induce BMSC chondrogenic differentiation, culture medium is traditionally supplemented with a variety of molecules said to be “chondrogenic”. While many pro-chondrogenic compounds have been extensively reported and used in differentiation assays^3,4^, laboratory-generated cartilage tissue does not yet yield structural and functional properties equivalent to native cartilage^2^.

Kartogenin (KGN), a small heterocyclic non-protein compound, was identified from a screen of 22,000 compounds as the most promising small molecule for inducing BMSC chondrogenesis^5^. While the initial report was promising, human BMSC response to KGN was not contrasted against more commonly used chondrogenic molecules, such TGF-β1, but instead only compared to the KGN solvent carrier DMSO^5^. KGN is available from Sigma-Aldrich, making it an accessible potential alternative to TGF-β1 or other common chondrogenic growth factors. A subsequent study investigated the effects of TGF-β1 and KGN on bovine chondrocytes and observed only weak KGN potency, and reasoned that biological response would likely be dependent on cell phenotype and differentiation status^6^. In rat BMSC cultures TGF-β1, KGN, and BMP-7 work synergistically, with KGN alone having negligible chondrogenic potency^7^. However, we note that these rat BMSC cultures responded modestly to TGF-β1-only medium formulations^7^, and that this differs from the expected significant response of human BMSC to TGF-β1 supplemented medium^8^. Critical benchmarking of KGN potency relative to canonical inductive factors, such as TGF-β1, in human BMSC cultures remains incomplete.

**Figure 1:**
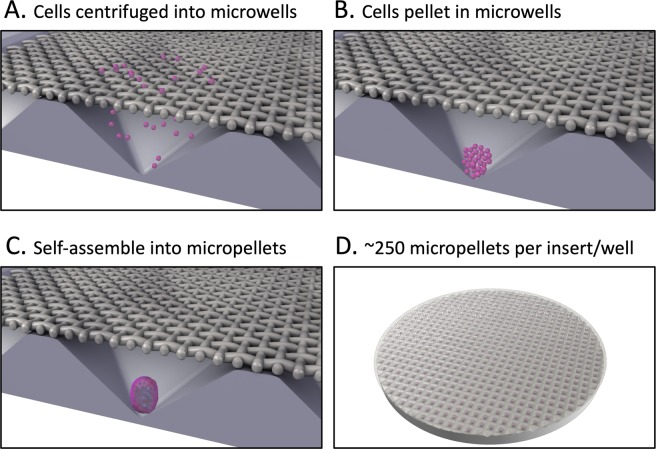
The *Microwell-mesh* platform was used for high throughput manufacture of cartilage micropellets. *Microwell-mesh* discs were inserted into tissue culture plastic wells and the system was sterilized prior to use in cell culture. Each microwell was 2×2 mm by 0.8 mm deep. (**A**) Cells were added to the tissue culture wells and forced to aggregate at the bottoms of microwells via centrifugation. (**B**) Centrifugation pelleted cells to the bottom of microwells. (**C**) Cells self-assembled into micropellets within 24 hours and were retained by the mesh. (**D**) Full view of a *Microwell-mesh* insert with ~250 micropellets; these inserts fit snuggly into the bottom of 6 well plates. Images were generated by abpLearning (www.medical-animations.com, Australia) using SoftImage (Autodesk, Montreal, Canada) and gifted to the Doran Laboratory.

The classic BMSC chondrogenic differentiation model is the pellet culture^9^, and this model was used to characterise BMSC response to KGN in the original publication^5^. Individual pellet cultures are typically formed from 2–5×10^5^ BMSC, yielding a tissue 2–3 mm in diameter^9,10^. These large-diameter tissues suffer from radial diffusion gradients, and as a result radial matrix and cell heterogeneity is common^8,10^. The histological characterisation of the pellet cultures used in the original study reveal radial heterogeneity for cartilage-like matrix staining, including type II collagen, aggrecan, and Safranin O (Fig. 1D in^5^). We reasoned that a source of inconsistency in reports regarding KGN potency in the original report^5^, and subsequent studies^6,7^, could be complicated by the heterogeneity in pellet culture readout used in the original assay^5–7^. Our team developed a microwell platform with the aim of being able to manufacture hundreds of smaller diameter pellets (*micro*pellets, 5×10^3^ cells each) that would yield more homogeneous cartilage-like tissue^8^. Use of more homogeneous micropellets should enable more reliable assessment of chondrogenic induction and characterisation of the relative potency of induction factors. The microwell platform (the *Microwell-mesh*) used to manufacture micropellets was described previously^8^. Given the potential of KGN, but lack of understanding of relative potency between KGN and TGF-β1 in human BMSC cultures, we sought to characterise KGN using a more homogeneous micropellet model. Herein we used the *Microwell-mesh* to manufacture hundreds of small diameter cartilage micropellets (5×10^3^ BMSC each) from human BMSC and evaluated the chondrogenic potency of TGF-β1 alone, KGN alone, and TGF-β1 + KGN. Chondrogenesis was evaluated based on relative matrix accumulation, histology and gene expression.

## Materials and Methods

### Isolation and culturing of human BMSC

As described previously^8^, bone marrow aspirates were collected from the iliac crest of consenting healthy adult volunteer donors. Mater Health Services Human Research Ethics Committee and the Queensland University of Technology Human Ethics Committee (1000000938) approved these collections. All methods were carried out in accordance with relevant guidelines and regulations. Bone marrow aspirate was diluted 1:1 with 2 mM EDTA in PBS, and overlayed on 15 mL of Ficoll-Paque PLUS (GE Healthcare). The solution was centrifuged for 30 min at 400 x g after which interface cells were collected, washed, and resuspended in low glucose Dulbecco’s Modified Eagle’s Medium containing 10% fetal bovine serum (FBS; Thermo Fisher Scientific), 10 ng/mL fibroblast growth factor-1 (FGF-1; Peprotech) and 100 U/mL penicillin/streptomycin (PenStrep; ThermoFisher). The cells were seeded in Nunc T175cm^2^ flasks (ThermoFisher) and incubated overnight in a normoxic incubator (20% O_2_) with 5% CO_2_ at 37 **°**C. The following day, the medium was aspirated, and fresh medium was added. Adherent cells were further passaged and seeded at ~1500 cells/cm^2^ in T175 cm^2^ flasks, and expanded in a hypoxic incubator (2% O_2_, 5% CO_2_) and medium was exchanged twice weekly. When cells were 80–90% confluent, they were passaged with 0.25% Trypsin/EDTA (ThermoFisher) and re-seeded as above.

### Fabrication and preparation of microwell-mesh platform

The Microwell-mesh was fabricated as shown in Fig. 1 (described here^8^). Briefly, a ~4 mm layer of polydimethylsiloxane (PDMS, Dow Corning) was cast on a polystyrene negative template with an inverted microwell pattern (microwells measured 2 mm ×2 mm with a depth of 0.8 mm^8^). A wad punch was utilized to generate round discs from sheets of PDMS. A nylon mesh (36 µm square pore openings, Part Number CMN-0035; Amazon.com) was bound over the microwell openings with silicone glue (Selleys Aquarium Safe). Discs were anchored into Nunc 6-well plates (ThermoFisher) with silicone glue. The Microwell-mesh was sterilised in 70% ethanol solution for minimum 30 minutes, and rinsed 3X with phosphate buffered saline (PBS; ThermoFisher). Immediately prior to cell seeding, a sterile 5% Pluronic solution (F-127 Pluronic, Sigma-Aldrich) in PBS was added to wells for 5 minutes to render the PDMS surface non-adhesive and promote cell aggregation^11,12^. Wells were rinsed 3X with PBS to remove excess Pluronic.

### Chondrogenic induction medium

BMSC were trypsinised and resuspended in a chondrogenic medium composed of HG-DMEM, 1X GlutaMax (ThermoFisher), 100 nM dexamethasone (Sigma-Aldrich), 200 µM ascorbic acid 2-phosphate (Sigma-Aldrich), 100 µM sodium pyruvate (ThermoFisher), 40 µg/mL L-proline (Sigma-Aldrich), 1% ITS-X (ThermoFisher) and 100 U/mL PenStrep (ThermoFisher). Either 10 ng/mL TGF-β1 (PeproTech), 10 µM KGN (Sigma-Aldrich), or both were added.

### Generation of micropellet and macropellet cultures

To eliminate air bubbles retained in microwells, 3 mL of cell-free chondrogenic medium was added to each well, and plates were centrifuged for 5 min at 2000 x g (Fig. 1A). Each well was seeded with 1.2×10^6^ BMSC in 1 mL of chondrogenic medium, yielding approximately 5000 cells per microwell. Classic pellet cultures were used in validation studies, and to delineate these from micropellets (5×10^3^ BMSC) these cultures are referred to as “*macro*pellets (2×10^5^ BMSC)”. These macropellet cultures were generated by seeding 2×10^5^ BMSC in 1 mL of induction medium in 96-deep well V-bottom plates (Corning). V-bottom plates were also coated with 5% Pluronic solution (Sigma-Aldrich) and rinsed well with PBS prior to cell seeding. Both plate types were centrifuged for 5 min at 150 x g to aggregate BMSC at the bottom of wells^8,11^. Cultures were maintained at 2% O_2_ plus 5% CO_2_ in a 37 °C incubator. Medium was exchanged every other day. A portion of the exchanged medium was collected and stored at −30 °C for future glycosaminoglycan (GAG) quantification. After the experimental period, the mesh was peeled from the PDMS discs to enable harvest of micropellets.

### Quantification of glycosaminoglycans (GAG) and DNA

Tissues were digested overnight in papain at 60 °C (1.6 U/mL Papain, 10 mM L-cysteine; both Sigma-Aldrich). GAG in the digested tissues and in medium samples was quantified utilising the 1,9-dimethylmethylene blue (DMMB, Sigma-Aldrich) assay^10^. A standard curve was generated using chondroitin sulfate sodium salt from shark cartilage (Sigma-Aldrich). A Quant-iT PicoGreen dsDNA assay kit (ThermoFisher) was used to estimate DNA content in micropellets.

### Histology and immunohistochemistry

Day 7 and 14 micropellets were harvested and fixed in 4% paraformaldehyde (PFA) for 20 min and frozen in Tissue-Tek OCT compound (Sakura Finetek). Samples were cryosectioned at 7 µm (Leica Cryostat CM1850, Leica) and captured on poly-lysine coated slides (ThermoFisher). Prior to staining, the sectioned tissues were again fixed with 4% PFA for 15 min then rinsed with PBS. Sections stained with 1% Alcian blue (Sigma-Aldrich) in 3% acetic acid (pH 2.5) for 30 min. Slides were rinsed with water, followed by counterstaining with Nuclear Fast Red (5 min). Slides rinsed with water and mounted (CC/mount, Sigma-Aldrich) for imaging. Immunohistology was performed for type I, type II and type X collagens. Sections were treated with hyaluronidase (2 U/mL, Sigma-Aldrich) for 30 min at 37 °C. Slides were washed 2X with 0.025% Triton X-100/PBS then blocked with 10% normal goat serum (Invitrogen) at RT for 1 h. Primary antibodies (Abcam) raised against type I (1:800; ab6308), type II (1:100; ab34712) and type X collagen (1:100; AB58632) were diluted in 1% BSA/PBS and slides were incubated at 4 °C overnight. The following day, 0.025% Triton X-100/PBS was added to slides 2×5 min, followed by 0.3% H_2_O_2_ in 100% methanol for 15 min. Slides were rinsed 2X with PBS then incubated with secondary antibodies goat anti-rabbit IgG H&L (HRP; ab6721) or goat anti-mouse IgG H7L (HRP; AB97023; both 1:1000; Abcam) in 1% BSA/PBS for 60 min at RT, then washed 2X with PBS. The DAB kit chromogen (Abcam) was applied for 8 minutes then slides were rinsed well in water and mounted (CC/Mount, Sigma-Aldrich) for imaging.

### Quantitative real-time RT-PCR (qRT-PCR)

At Day 7 and 14, tissues were harvested and stored in Trizol at −80 °C (ThermoFisher, as manufacturer’s per protocol). A DNase I digest (Zymogen) was performed. RNA was reverse-transcribed using the SuperScript III First-Strand Synthesis System for qRT-PCR (ThermoFisher). RNA concentration was quantified with a NanoDrop Lite spectrophotometer (ThermoFisher). The qRT-PCR mastermix included 2X SYBR Green PCR Master Mix (Applied Biosystems), 200 nM of the forward and reverse primers, RNase-free water and 1 µL of sample cDNA. The 5 µL reactions were run in triplicate in a 384 well plate inside a Viia7 Real Time PCR System (Applied Biosystems). The initial cycle was 50 °C for 2 min, then 95 °C for 10 min, followed by 40 cycles of 95 °C for 15 seconds and 60 °C for 1 min. The specificity of products was confirmed by melt curve analysis. Table 1 lists primer set information. Primers for *ACAN*, *COL1A1*, *COL2A1*, *BGLAP* and *VCAN*^13^, *RPLPO* and *COL10A1*^14^, *RUNX2*^15^ and *IHH*^16^ were as previously published in the literature. Primers for *SOX9* were designed using Primer3Plus software^17^.

**Table 1:** Primers used for RT-qPCR for human genes.

Gene	Sequence (5′ to 3′)	Amplicon size (bp)
*RPLPO*	F: TGTGGGCTCCAAGCAGATGCA R: GCAGCAGTTTCTCCAGAGCTGGG	137
*ACAN*	F: TCGAGGACAGCGAGGCCR: TCGAGGGTGTAGCGTGTAGAGA	85
*COL1A1*	F: CAGCCGCTTCACCTACAGC R: TTTTGTATTCAATCACTGTCTTGCC	83
*COL2A1*	F: GGCAATAGCAGGTTCACGTACA R: CGATAACAGTCTTGCCCCACTT	79
*COL10A1*	F: ACTCCCAGCACGCAGAATCCA R: TGGGCCTTTTATGCCTGTGGGC	132
*RUNX2*	F: GGAGTGGACGAGGCAAGAGTTT R: AGCTTCTGTCTGTGCCTTCTGG	133
*SOX9*	F: ACTCCTCCTCCGGCATGAG R: GCTGCACGTCGGTTTTGG	102
*RUNX1*	F: CCACCTACCACAGAGCCATCAA R: TTCACTGAGCCGCTCGGAAAAG	122
*BGLAP*	F: GAAGCCCAGCGGTGCA R: CACTACCTCGCTGCCCTCC	70
*VCAN*	F: TGGAATGATGTTCCCTGCAA R: AAGGTCTTGGCATTTTCTACAACAG	98

### Data collection and statistical analysis

Statistical analysis was done using GraphPad Prism. Data were analysed using two-way ANOVA and statistical significance was determined using Tukey’s test. Experiments were replicated with 3 different BMSC donors (referred to here as Donor 1, Donor 2, and Donor 3). Results are represented for all donors, with each figure showing mean ± SD, n = 4 replicate cultures, P < 0.05 unless otherwise noted.

### Ethical approval

Bone marrow aspirates were collected from consenting healthy adult volunteer donors. Ethics was approved by the Mater Health Services Human Research Ethics Committee and the Queensland University of Technology Human Ethics Committee (1000000938).

### Informed consent

Written consent was collected from bone marrow donors, and consent data is retained by the Mater Health Services Human Research Ethics Committee.

## Results

### Growth of micropellets in the Microwell-mesh system

**Figure 2:**
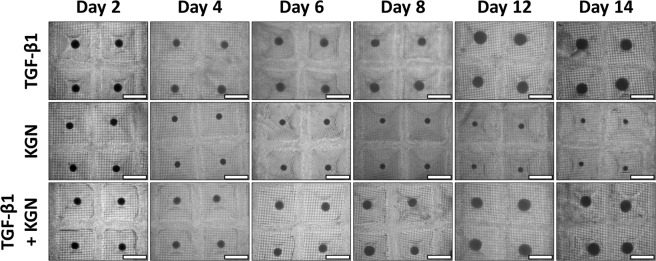
BMSC Donor 1. Microscope images of pellets over a 14-day culture period. Micropellets were imaged throughout the differentiation period. At Day 14, micropellets in the two groups containing TGF-β1 were larger than those in the KGN alone group. Scale bar = 1 mm. Replicate images from BMSC donors 2 and 3 are shown in Supplementary Figs. 1 and 2.

Each micropellet was initially formed from approximately 5×10^3^ human BMSC each, with approximately 250 replicate micropellets produced in each *Microwell-mesh* disc. Over 14 days of culture, micropellets cultured in TGF-β1 and TGF-β1 + KGN increased in diameter, while KGN micropellets decreased in size (Fig. 2, Supplementary Figs. 1 and 2).

### Glycosaminoglycan production by micropellets is higher with TGF-β1 supplementation

**Figure 3:**
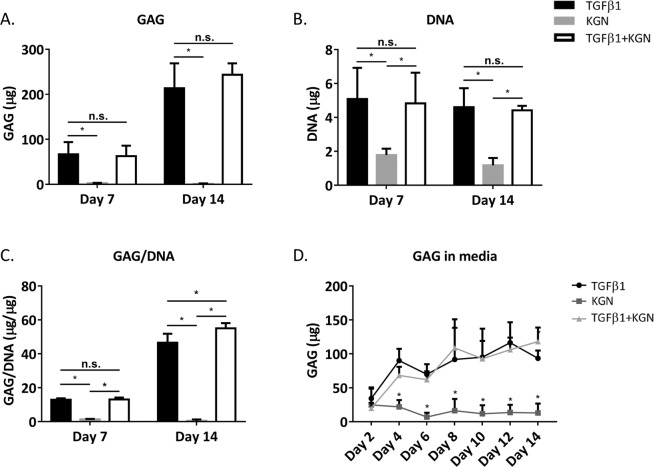
BMSC Donor 1. GAG and DNA quantities in micropellets, GAG/DNA, and GAG secreted to media. (**A**) Quantities of GAG in micropellets at Day 7 and Day 14. (**B**) DNA quantities in micropellets. (**C**) GAG normalized to DNA in micropellets. (**D**) Quantification of GAG secreted to the media by micropellets over a 14-day culture period. For A-C, mean ± SD, n = 4, P < 0.05; for D, mean ± SD, n = 6, P < 0.05. Refer to Supplementary Figs 3 and 4 for replicate donor data sets.

Glycosaminoglycan (GAG) production by the three BMSC donors differed in response to TGF-β1 and KGN, but there was a consistent pattern indicating that the presence of TGF-β1 was required to maximise micropellet GAG content. For Donor 1, micropellets cultured in TGF-β1 and TGF-β1 + KGN yielded similarly elevated GAG content, while the KGN group produced almost no GAG (Fig. 3A). In contrast, Donor 2 BMSC generated more GAG in response to TGF-β1 alone relative to TGF-β1 + KGN or KGN alone (Supplementary Fig. 3A). Like Donor 1, Donor 3 yielded similar GAG content in response to either TGF-β1 alone or TGF-β1 + KGN (Supplementary Fig. 4A). In all cases, KGN alone yielded significantly less GAG than any culture condition that contained TGF-β1.

Micropellet DNA content was similar in TGF-β1 alone and TGF-β1 + KGN culture conditions for all three BMSC donors (Fig. 2B, and Supplementary Figs. 3B and 4B). Similarly, the micropellet DNA content of all three BMSC donors was approximately 50% lower in KGN cultures, relative to the TGF-β1 alone and TGF-β1 + KGN culture conditions. GAG quantities were normalized with DNA values (Fig. 3C, Supplementary Figs 3C and 4C). As DNA content was similar in the TGF-β1 and TGF-β1 + KGN groups for all donors, GAG/DNA was influenced by differences in donor-specific GAG production in response to either TGF-β1 alone or TGF-β1 + KGN. Finally, the GAG content in the medium at all medium exchange timepoints was quantified for all culture conditions. Micropellets formed from Donor 1 BMSC yielded similar quantities of secreted GAG in the TGF-β1 alone and TGF-β1 + KGN conditions, and this quantity was markedly greater than in KGN alone cultures. Micropellets formed from Donor 2 BMSC yielded the greatest secreted GAG in the TGF-β1 alone culture, then TGF-β1 + KGN, and then KGN alone. Micropellets formed from Donor 3 BMSC yielded greater secreted GAG in response to TGF-β1 + KGN across most of the medium collection timepoints. For all BMSC donors, secreted GAG quantities were the lowest in cultures where the medium was supplemented with KGN alone. In all cases, the pattern of relative GAG secretion (GAG detected in the medium) paralleled the pattern of GAG quantity measured in the micropellet tissues.

### Distribution of extracellular matrix molecules in micropellets

**Figure 4:**
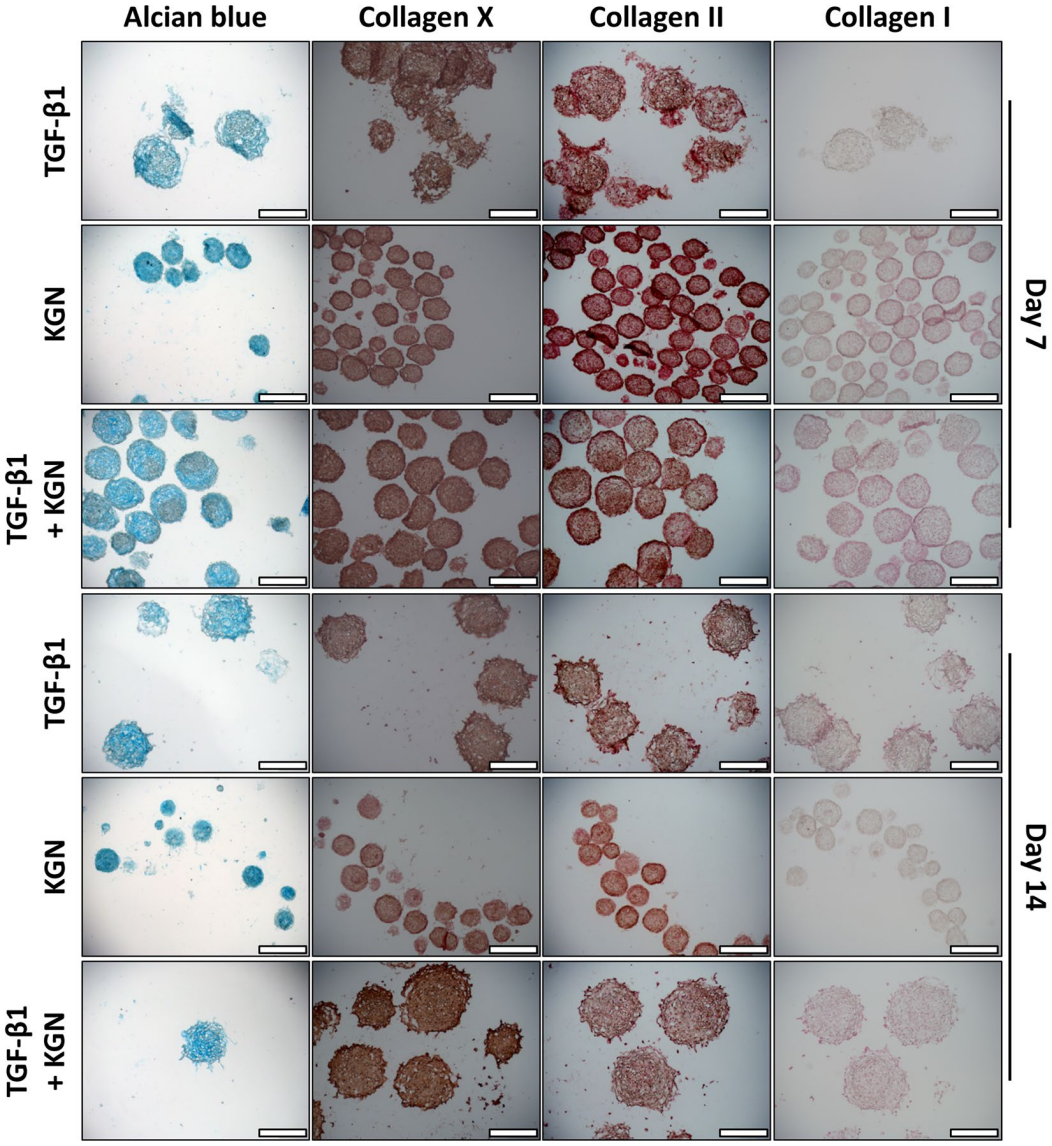
BMSC Donor 1. Histology: Alcian blue and type X, II and I collagen. Alcian blue (first column) was used to stain sections of micropellets. Type X collagen immunohistochemical staining of sections of micropellets is shown in the second column. The third column shows type II collagen immunohistochemical staining of sections of micropellets. The final column shows type I collagen immunohistochemical staining of sections of micropellets. Scale bar = 400 μm. Refer to Supplementary Figs 5 and 6 for replicate donor data sets.

Alcian blue staining revealed cartilage-like matrix accumulation in micropellets from all three groups (Fig. 4). KGN micropellets were smaller in size than micropellets from the other two groups, and these tissues had less obvious lacunae development. Collagen X staining was present in all conditions and time points, with greatest staining observed in the TGF-β1 + KGN Day 14 micropellets (Fig. 4). Collagen II staining was prevalent in all conditions and at all time points (Fig. 4). Collagen I staining was present across all conditions and time points (Fig. 4), although collagen I staining appeared to be weaker than collagen II staining. Donor replicates are shown in Supplementary Figs 5 and 6.

### Gene expression of micropellets

**Figure 5:**
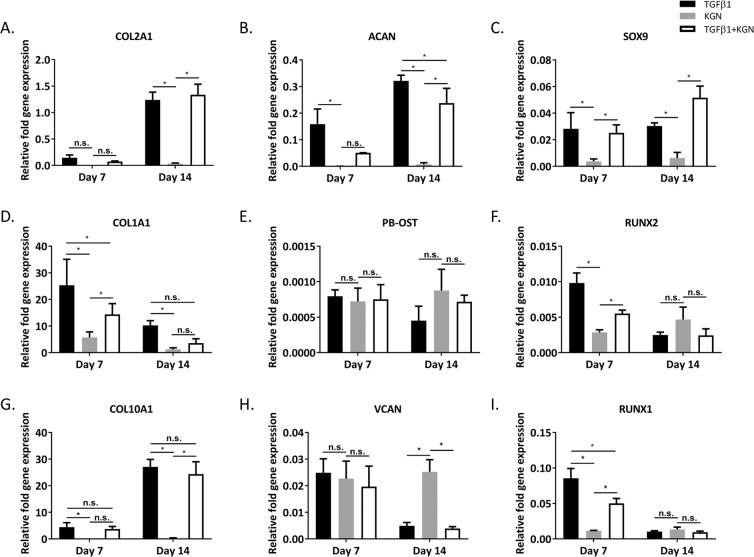
BMSC Donor 1. qRT-PCR analysis of cartilage-like tissues generated from BMSC. Increased expression of chondrogenic markers was observed in both TGF-β1 conditions compared to KGN alone. Expression levels are shown relative to *RPLPO* gene expression (plotted as mean ± SD, n = 4, P < 0.05). Refer to Supplementary Figs. 7 and 8 for replicate donor data sets.

The expression of chondrogenic marker genes *COL2A1* (Fig. 5A), *ACAN* (5B), and *SOX9* (5 C) was significantly higher in the two TGF-β1 conditions compared to the KGN alone condition. This pattern was observed across all three donors. Osteogenic markers *COL1A1* (5D), *PB-OST* (5E), *RUNX2* (5 F) of the KGN alone group were not significantly different from the two TGF-β1 groups at Day 14, except for *COL1A1* where the TGF-β1-only condition was significantly higher than the KGN alone condition. Inducing chondrogenesis of BMSC using TGF-β1 typically leads to hypertrophy of the cells by activating an intrinsic differentiation program reminiscent of endochondral bone formation^2^. Expression of the hypertrophy marker *COL10A1* was indeed higher in the TGF-β1 condition than in the KGN alone group, across all three donors. Donor replicates are shown in Supplementary Figs. 7 and 8.

### BMSC macropellet culture response to TGF-β1 is greater than KGN

For direct comparison to common methods used in the field, and methods used in the original KGN publication^5^, macropellets were manufactured from 2×10^5^ human BMSC each. This evaluation was also executed to ensure that poor response to KGN was not an artefact of the micropellet model. Macropellets were cultured under four different medium conditions: (1) KGN alone, (2) KGN in combination with chondrogenic media (without TGF-β1), (3) chondrogenic media without TGF-β1, or (4) chondrogenic media with TGF-β1. Following 14 days of culture, the macropellets cultured with TGF-β1 (condition 4) exhibited the largest diameters (Supplementary Fig. 9). The macropellets in the other three groups, none of which contained TGF-β1, remained a similar size or became smaller over the culture period. Similarly, GAG quantification (Supplementary Fig. 10A) showed significantly more GAG accumulation in the TGF-β1 group at Day 14 than in any of the other three conditions. No significant difference was observed between the other three groups at Day 14, and there was no significant difference among all four groups at Day 7. In contrast, significantly less DNA was detected at Day 7 and 14 in the KGN alone group (condition 1) than in other groups (Supplementary Fig. 10B). When GAG was normalized to DNA (Supplementary Fig. 10C), the TGF-β1 group had GAG/DNA values 2–3 times that of the other three conditions at Day 14. GAG in media was also quantified (Supplementary Fig. 10D), and TGF-β1 group values were significantly higher at some time points but not others. Alcian blue staining suggested that the Day 14 TGF-β1 condition had the highest accumulation of GAG, and the KGN alone condition had the lowest (Supplementary Fig. 11). These data paralleled our multiple observations using the microwell platform, suggesting weak chondrogenic induction of BMSC with KGN was independent of the pellet model used.

## Discussion

Engineering cartilage-like tissue from BMSC using current induction medium formulations remains a challenge^2^. The original KGN characterization was completed using cells from a single BMSC donor purchased from STEMCELL Technologies. The study did not contrast KGN directly against TGF-β1^5^, and this comparison has yet to be described with human BMSC. Here, the effects of 10 µM KGN supplementation were compared with the effects of supplementation with 10 ng/mL TGF-β1. In the original paper, 10 µM KGN was reported to induce greater expression of collagen II and aggrecan than other KGN concentrations^5^. Similarly, 10 ng/mL TGF-β1 has been reported to be a potent inducer of BMSC chondrogenesis in micropellet studies^8,10^.

Using the homogeneous micropellet culture system, which yields hundreds of replicate micropellets composed of 5000 cells each, KGN’s capacity to promote chondrogenesis was found to be substantially inferior to TGF-β1 in terms of GAG production, gene expression, and cartilage-like matrix accumulation. Even in the presence of induction molecules such as dexamethasone, BMSC chondrogenic induction in KGN-supplemented medium was unremarkable. Micropellets cultured in KGN-supplemented medium shrank over time, declined in DNA content, had little GAG content, and did not form lacunae structures normally associated with cartilage tissue. This pattern was consistent across cultures derived from all three human BMSC donors evaluated and was again replicated in macropellet cultures (200,000 BMSC each). When KGN was used in combination with TGF-β1, synergistic effects appeared minute and were not consistent across all BMSC donors. Hypertrophy gene expression was similarly elevated in TGF-β1 and TGF-β1 + KGN cultures, suggesting that KGN does not mitigate hypertrophy, a major obstacle in BMSC-based cartilage engineering strategies^2^.

While lower DNA quantities were observed in KGN micropellet cultures, this observation is unlikely to indicate KGN toxicity for two reasons. Firstly, the original paper reported no cytotoxic effects of KGN on human BMSC or chondrocytes at concentrations 10-fold higher than that used in this study. Secondly, in our study the TGF-β1 + KGN cultures had similar DNA quantities compared to the TGF-β1-only group. This suggests that reduced cell number was not caused by the presence of KGN, but rather by the absence of TGF-β1 in the KGN-only cultures. Macropellets cultured in KGN supplemented medium (with no other chondrogenic medium supplements) had the least DNA quantity. Again, when macropellets were cultured in medium with chondrogenic supplements plus or minus KGN the DNA content was similar, suggesting that KGN was not toxic. Paralleling micropellet results, the addition of TGF-β1 to the chondrogenic induction medium yielded the greatest DNA content in macropellets.

Direct comparisons between new molecular inducers of chondrogenesis and current canonical inducers, such as TGF-β1, are essential to guide future experimental studies. Our study demonstrates that KGN’s capacity to induce chondrogenesis in human BMSC is considerably weaker than that of TGF-β1. We validated this in both conventional pellet cultures (macropellets), and in more homogeneous micropellet cultures. While BMSC are a likely cellular input for future cartilage repair therapies, our results suggest that it is unlikely that KGN is sufficiently potent to function as an alternative to growth factors in promoting BMSC chondrogenesis. Consistent benchmarking of new compounds against current canonical inducers will add clarity to the literature, and help guide efficient research investment.

Finally, while KGN did not yield the expected results in our studies, we view this as an example of the replication challenges being experienced in a field where a range of different cell populations and model systems are being used to pursue a common goal; in this case, BMSC-mediated cartilage repair. KGN has been observed to drive a chondrogenic-like responses at a range of concentrations, but with variable outcomes depending on the species or tissue from which the cells were derived. For example, two papers used 1 µM KGN media supplementation to induce rat^18^ or rabbit^19^ BMSC chondrogenic induction. In the original Science publication, maximal human BMSC chondrogenic gene expression (aggrecan and collagen II) was observed with 10 µM KGN medium supplementation^5^. However, bioactivity was observed from 100 nM to 10 µM, with no toxicity at 100 µM^5^. In a different study, rat BMSC cultured in medium supplemented with 100 nM KGN significantly upregulated intracellular lubricin and extracellular lubricin, relative to control populations, but did not increase GAG production in response to KGN or TGF-β1 alone^7^. This response differs from most human BMSC studies where TGF-β1 alone increases GAG production^8–10,20^. While TGF-β1 or KGN alone did not yield positive outcomes, the authors found that a combination of TGF-β1, BMP-7 and KGN worked synergistically to drive rat BMSC chondrogenesis^7^. A potentially more relevant study published in 2019 reported that preconditioning of human umbilical cord-derived mesenchymal stromal cells in medium supplemented with 1 µM KGN enhanced their chondrogenic response to TGF-β3^21^. Similar to our observations with human BMSC, treatment of human umbilical cord-derived mesenchymal stromal cells with KGN alone was not chondrogenic. The authors concluded that KGN preconditioning likely improved chondrogenic differentiation of umbilical cord blood-derived mesenchymal stromal cells by committing them to a pre-cartilaginous stage with enhanced JNK phosphorylation and suppressed β-catenin^21^. Our team recently reported that exposing human BMSC to a single day of TGF-β1 yielded differentiation outcomes similar to BMSC exposed to TGF-β1 for 21 days^22^. This observation is specifically relevant to this paper for two reasons: (1) in the human BMSC micropellet model, a single day of TGF-β1 is sufficient to trigger chondrogenic induction, while 14 days of continuous KGN had minimal impact on human BMSC chondrogenesis; and (2) the mechanism by which a single day of TGF-β1 exposure drives BMSC differentiation appears to be fundamentally different than the mechanism by which KGN pre-conditioning of human umbilical cord-derived mesenchymal stromal cells promoted subsequent chondrogenesis in response to TGF-β3^21^. Given the opposing results in the literature, including possibly differing induction mechanisms, we recommend careful consideration of experimental design in KGN-based studies, including careful selection of the model system, species and tissue source, and consideration of KGN temporal dosing.

## Supplementary information

Supplementary Figure 1.

Supplementary Figure 2.

Supplementary Figure 3.

Supplementary Figure 4.

Supplementary Figure 5.

Supplementary Figure 6.

Supplementary Figure 7.

Supplementary Figure 8.

Supplementary Figure 9.

Supplementary Figure 10.

Supplementary Figure 11.

Supplementary Figures and Legends.
