# Supplementary figures and images for: Transforming growth factor-beta stimulates human bone marrow-derived mesenchymal stem/stromal cell chondrogenesis more so than kartogenin
Source: Sci Rep. 2020 May 20;10:8340. doi: 10.1038/s41598-020-65283-8 (PMC7239921; doi:10.1038/s41598-020-65283-8)

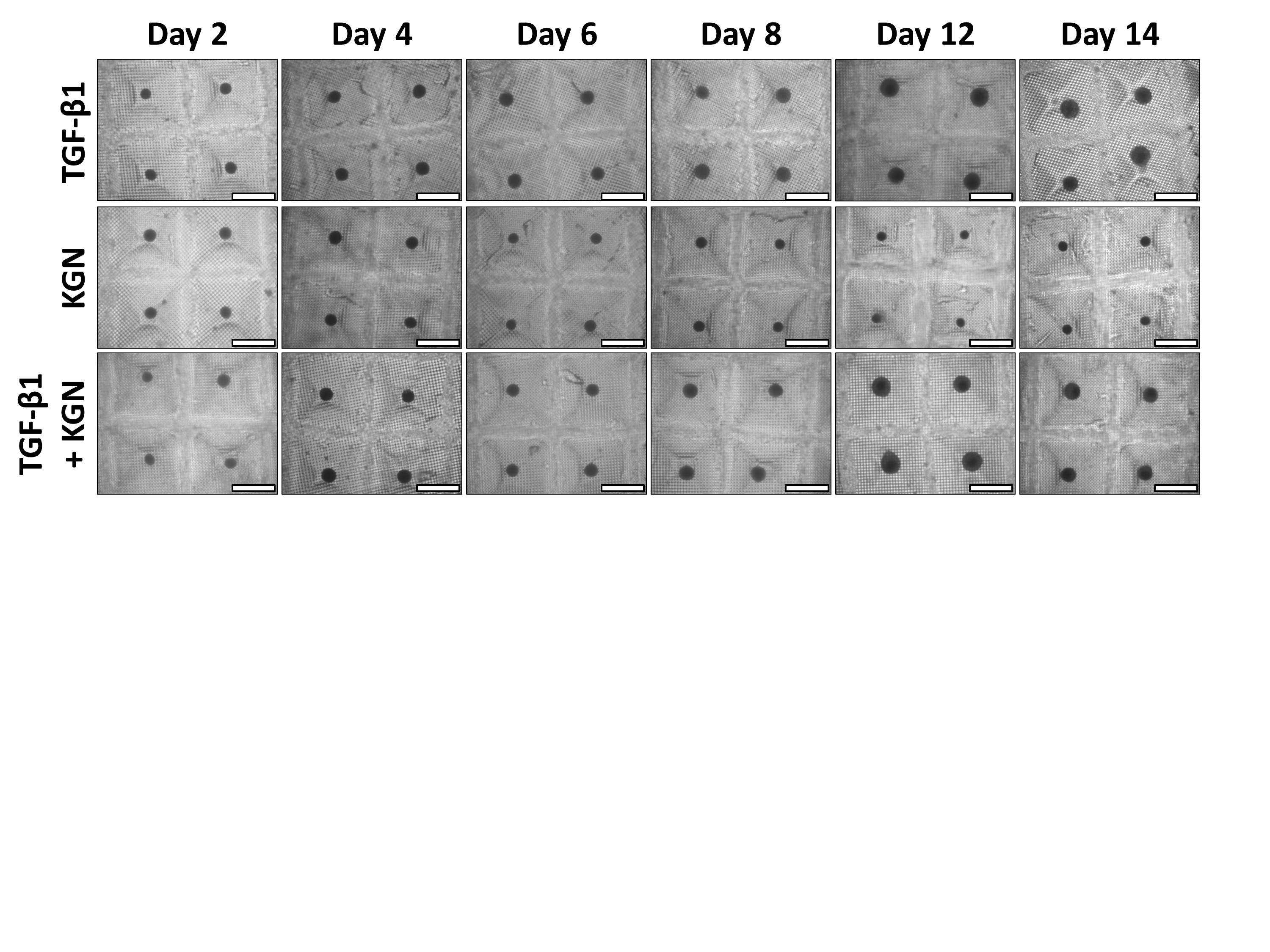

Supplement: Supplementary file 1 — Supplementary Figure 1. [file 41598_2020_65283_MOESM1_ESM.tif]

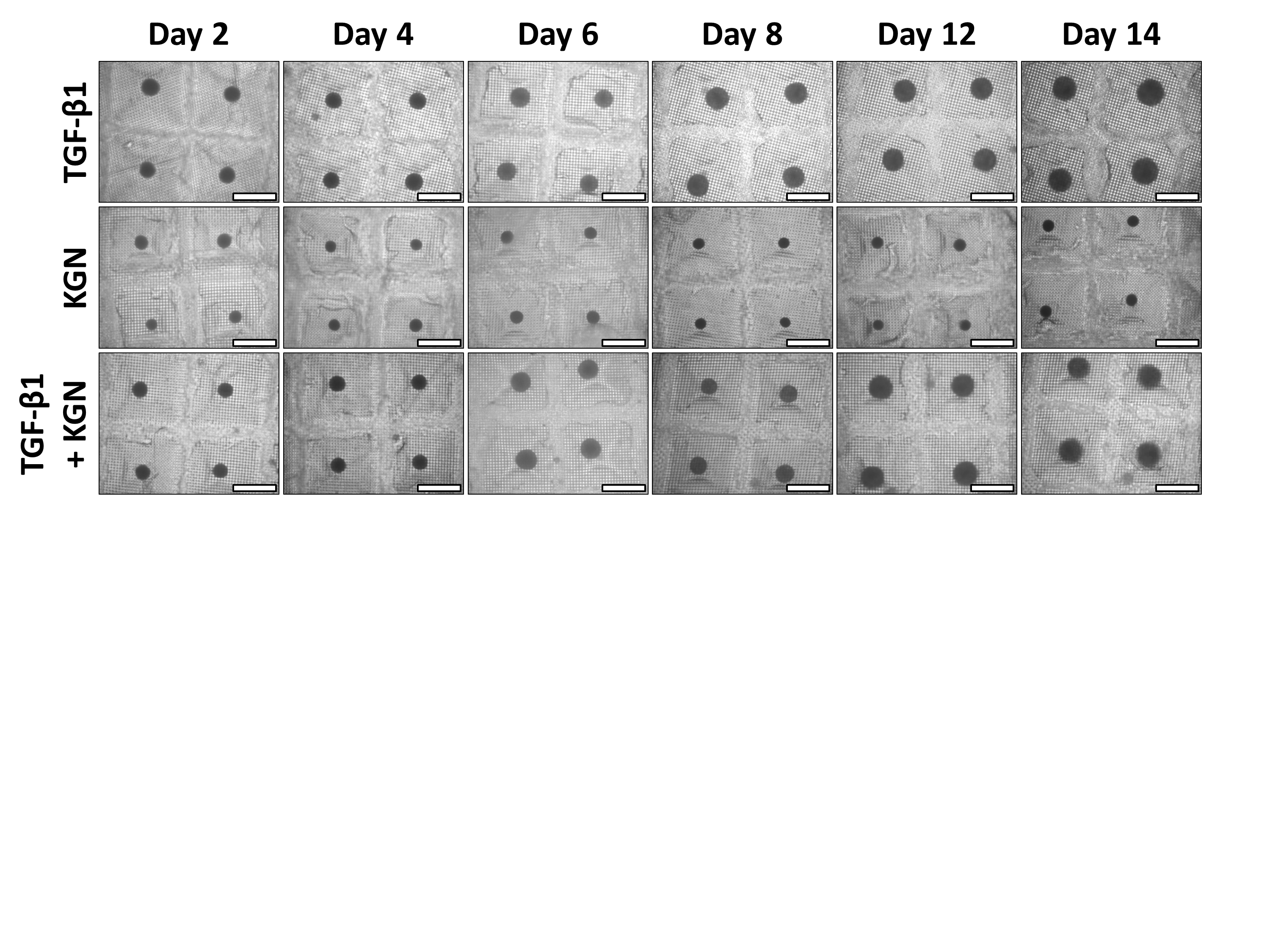

Supplement: Supplementary file 2 — Supplementary Figure 2. [file 41598_2020_65283_MOESM2_ESM.tif]

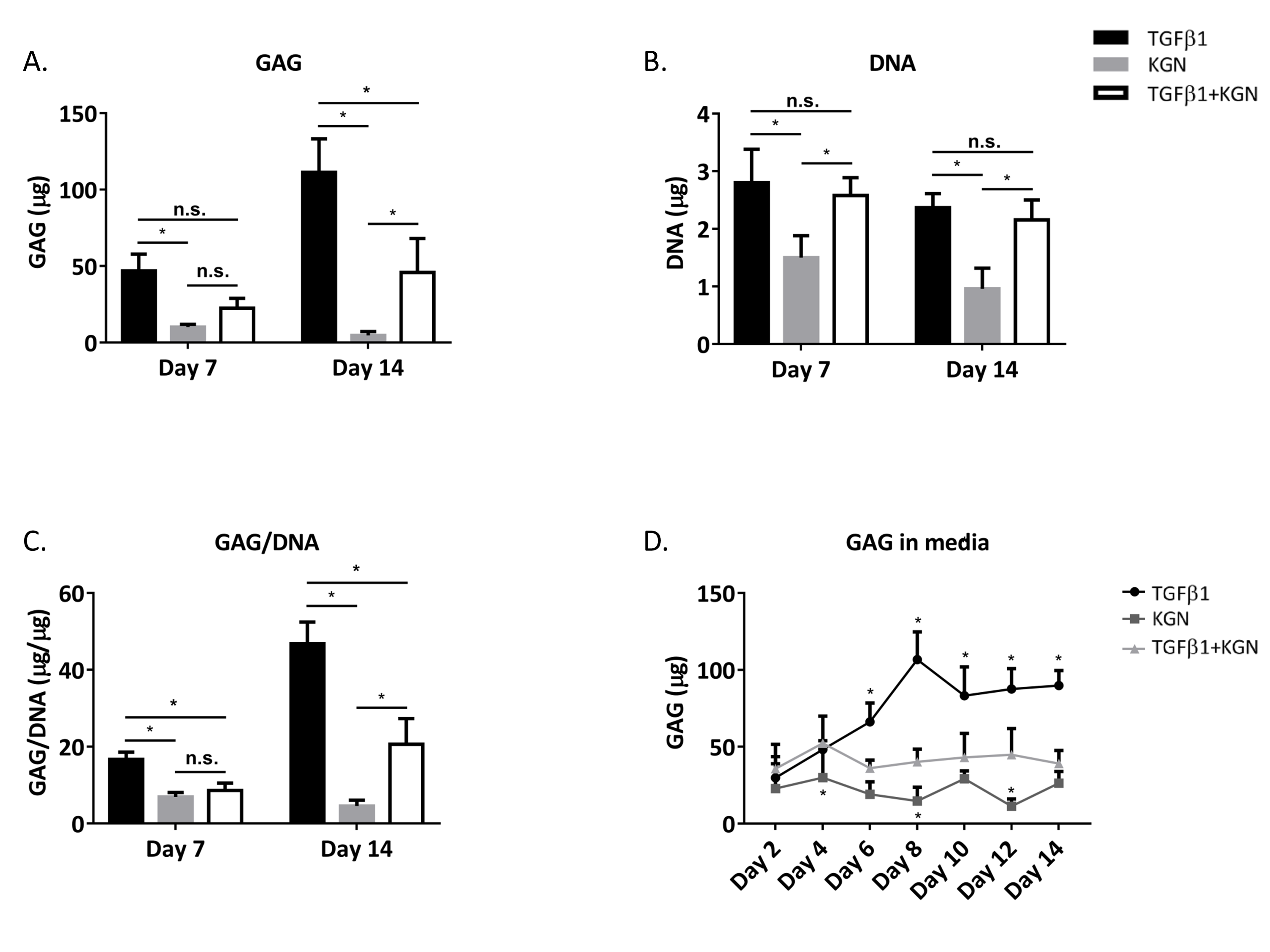

Supplement: Supplementary file 3 — Supplementary Figure 3. [file 41598_2020_65283_MOESM3_ESM.tif]

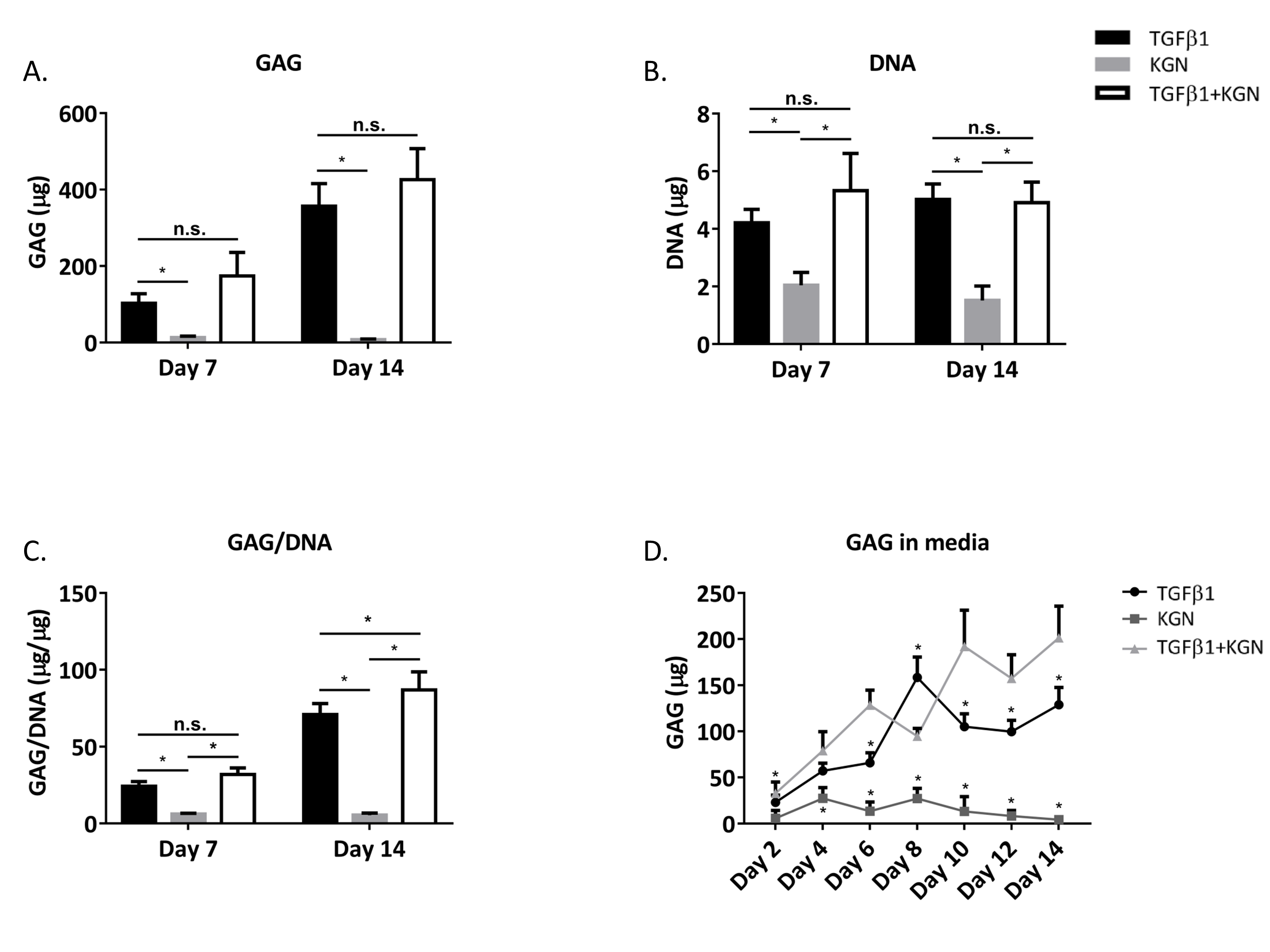

Supplement: Supplementary file 4 — Supplementary Figure 4. [file 41598_2020_65283_MOESM4_ESM.tif]

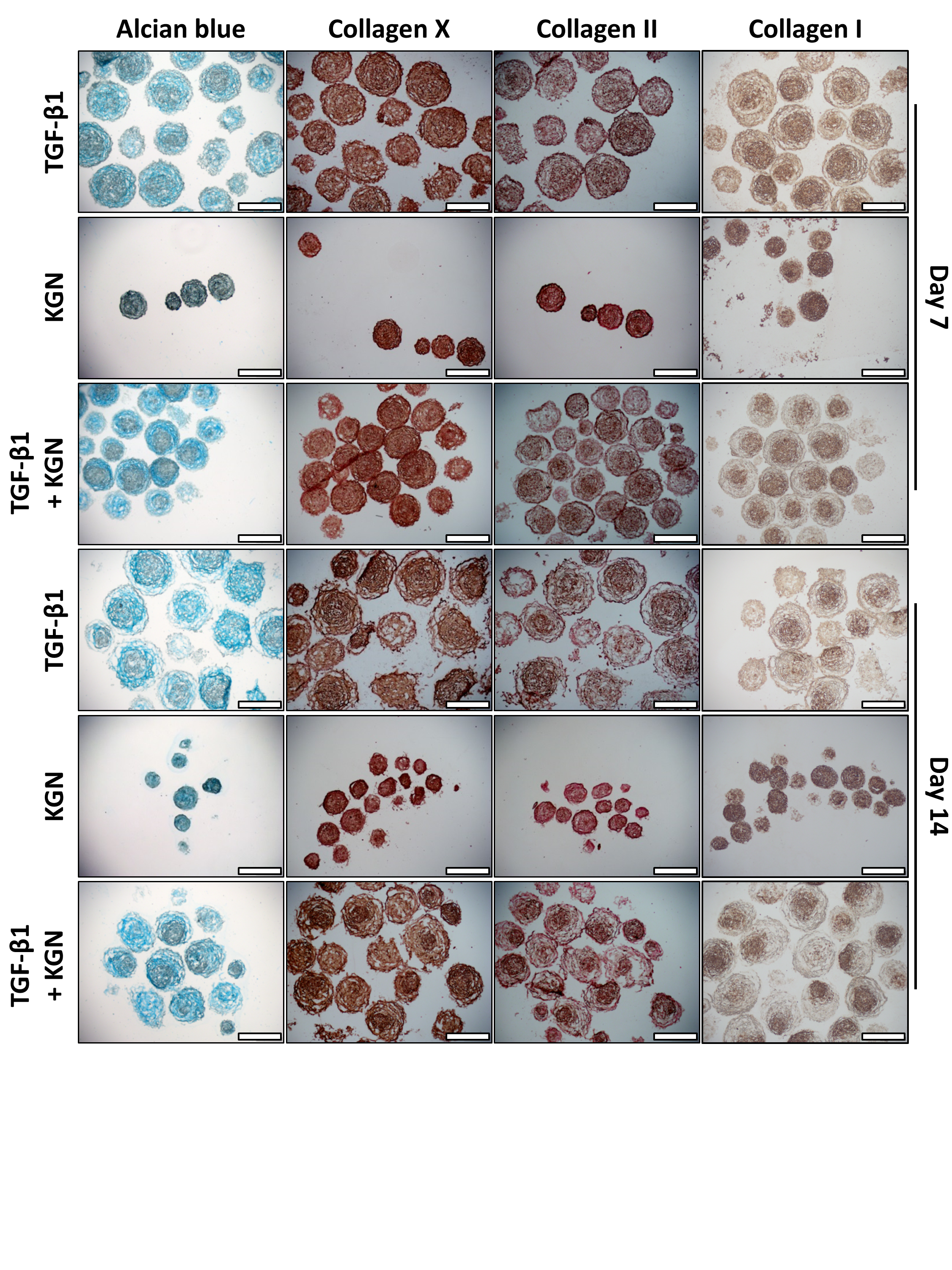

Supplement: Supplementary file 5 — Supplementary Figure 5. [file 41598_2020_65283_MOESM5_ESM.tif]

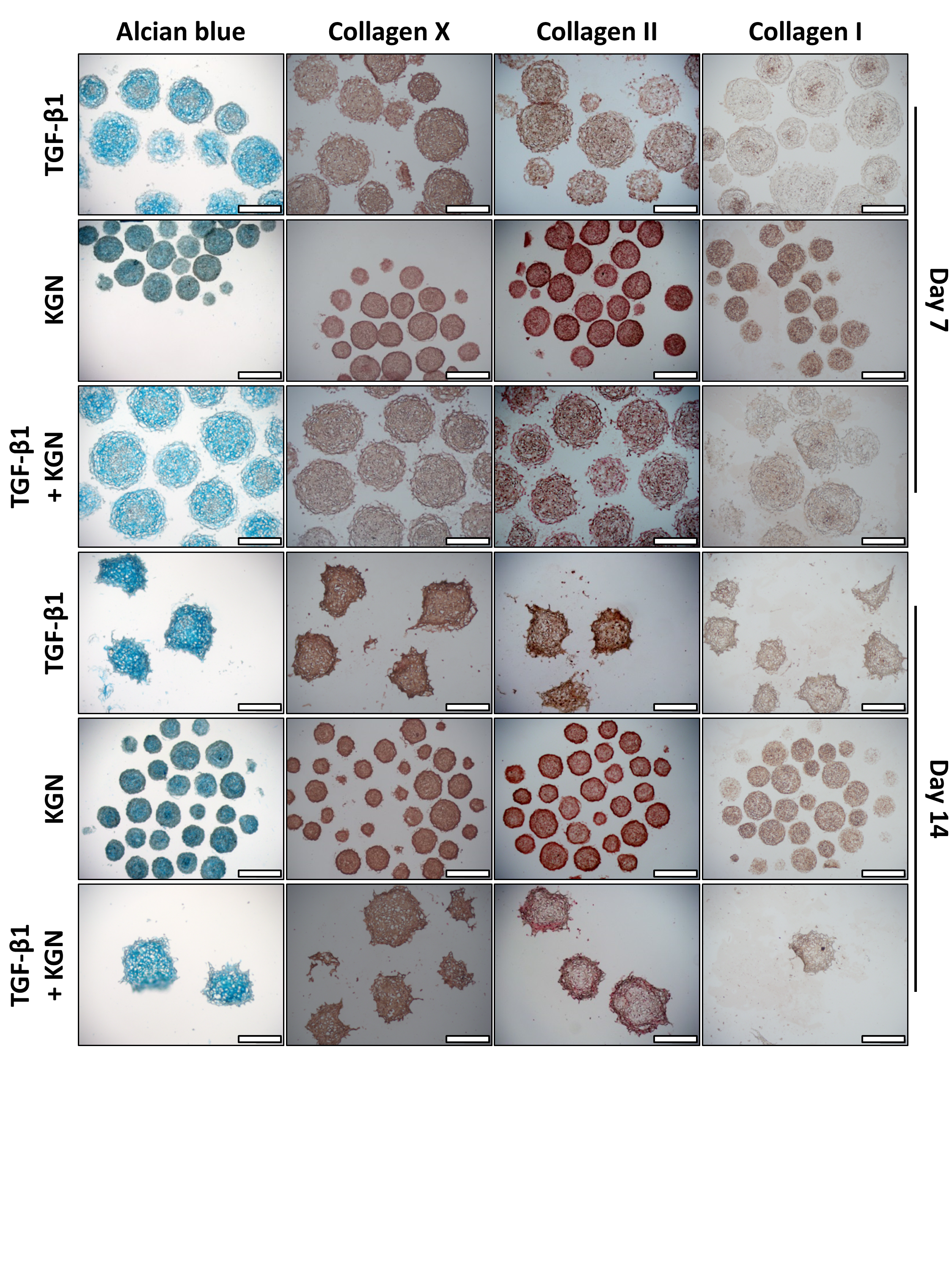

Supplement: Supplementary file 6 — Supplementary Figure 6. [file 41598_2020_65283_MOESM6_ESM.tif]

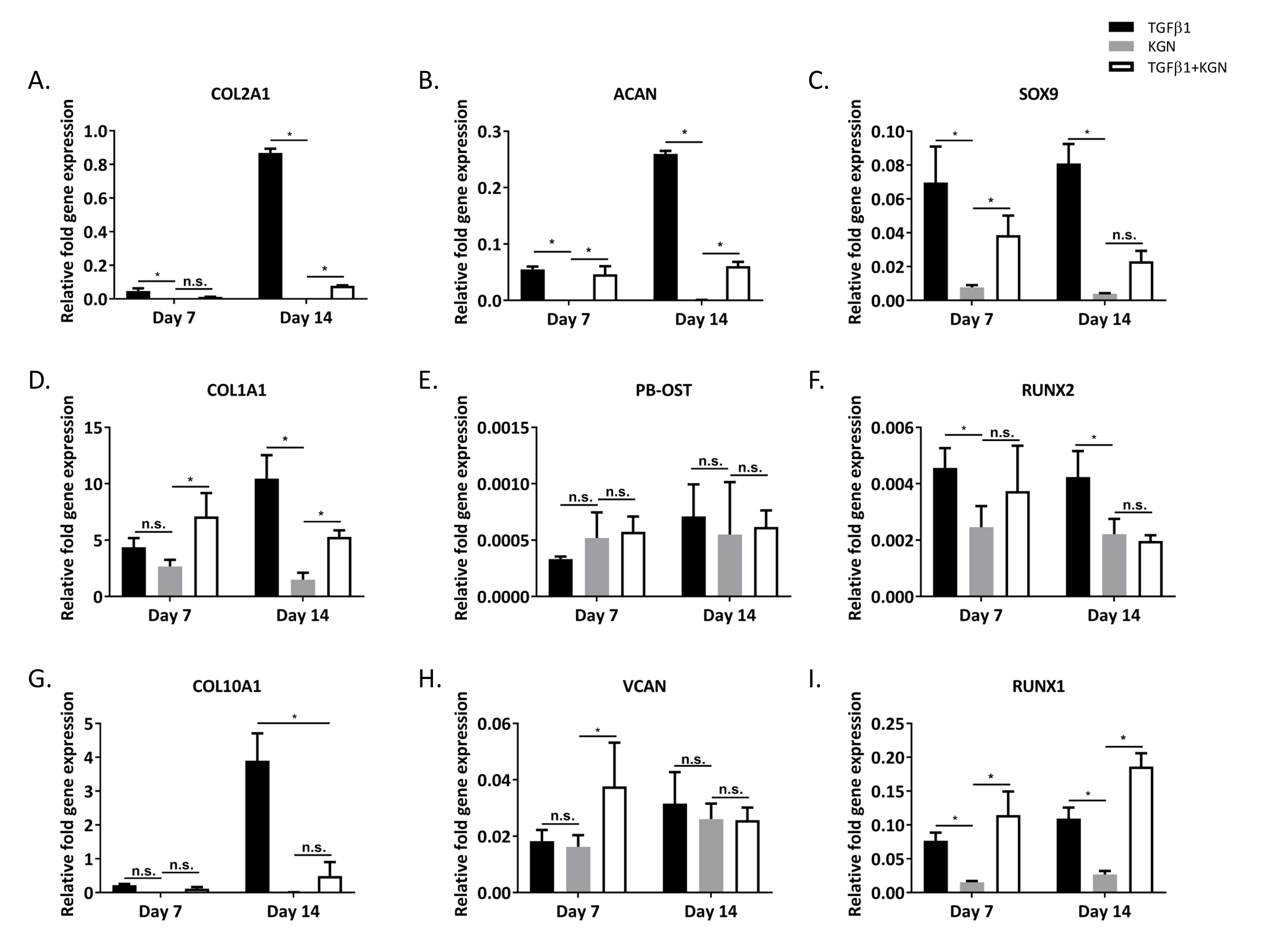

Supplement: Supplementary file 7 — Supplementary Figure 7. [file 41598_2020_65283_MOESM7_ESM.tif]

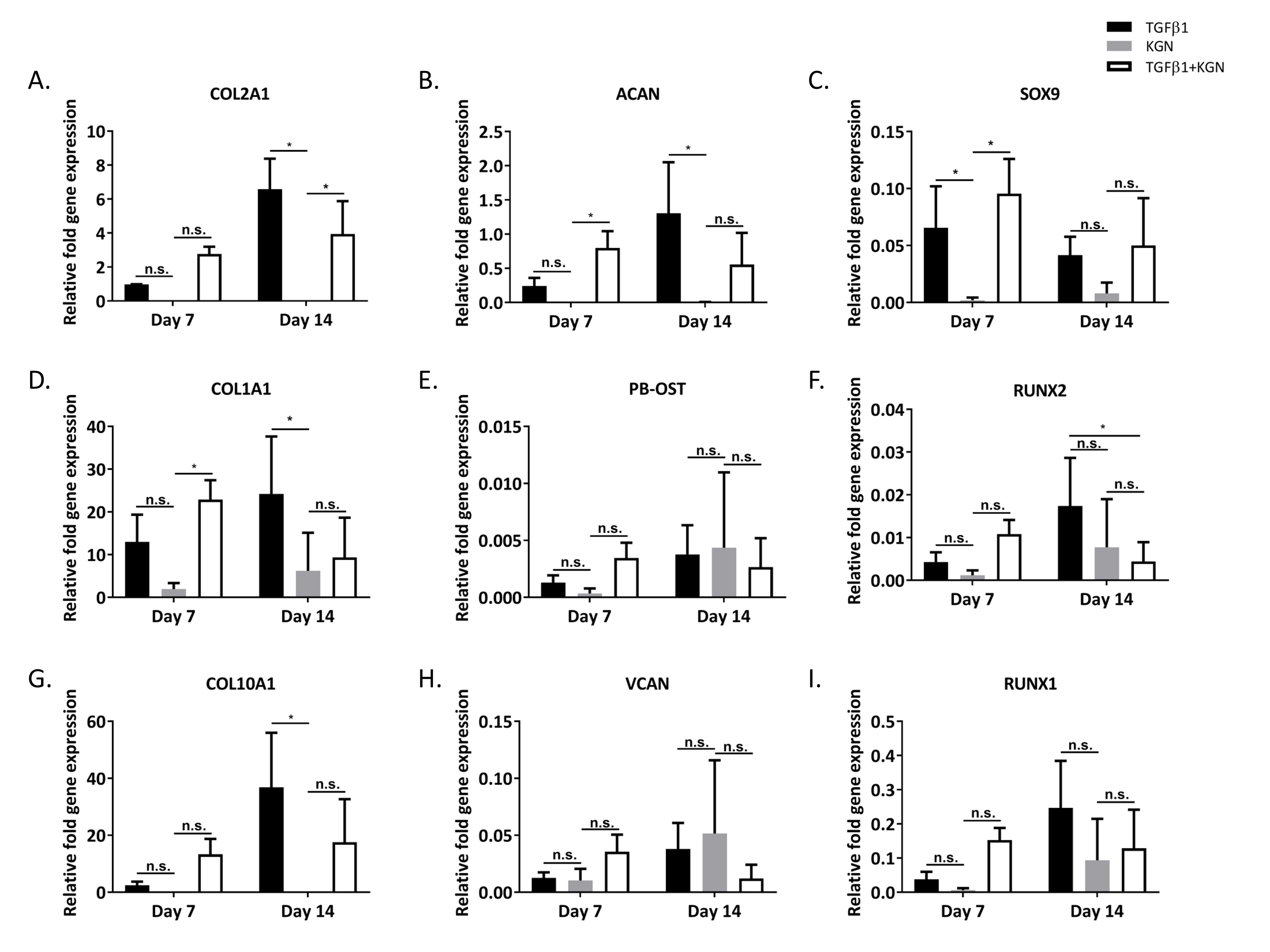

Supplement: Supplementary file 8 — Supplementary Figure 8. [file 41598_2020_65283_MOESM8_ESM.tif]

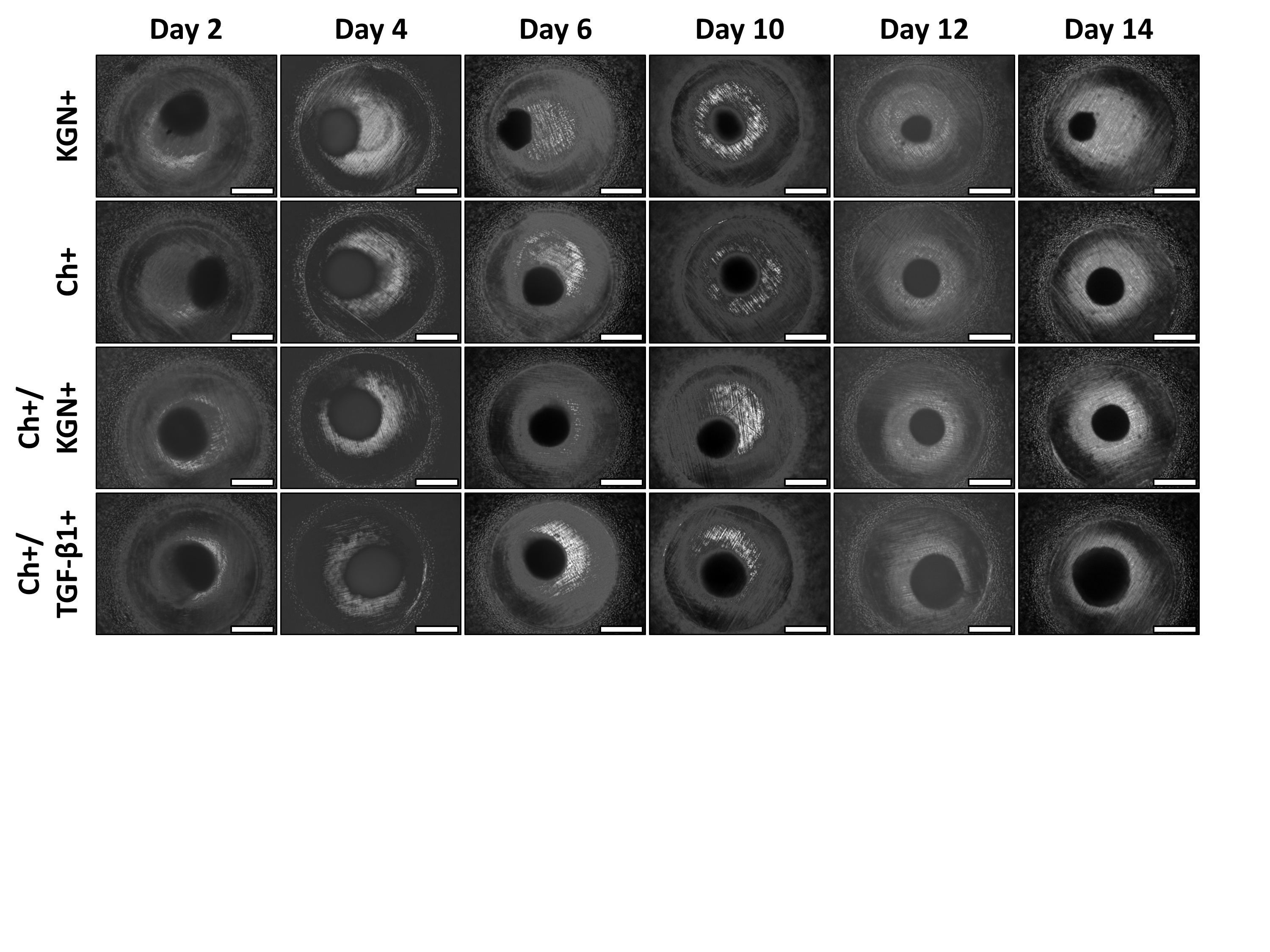

Supplement: Supplementary file 9 — Supplementary Figure 9. [file 41598_2020_65283_MOESM9_ESM.tif]

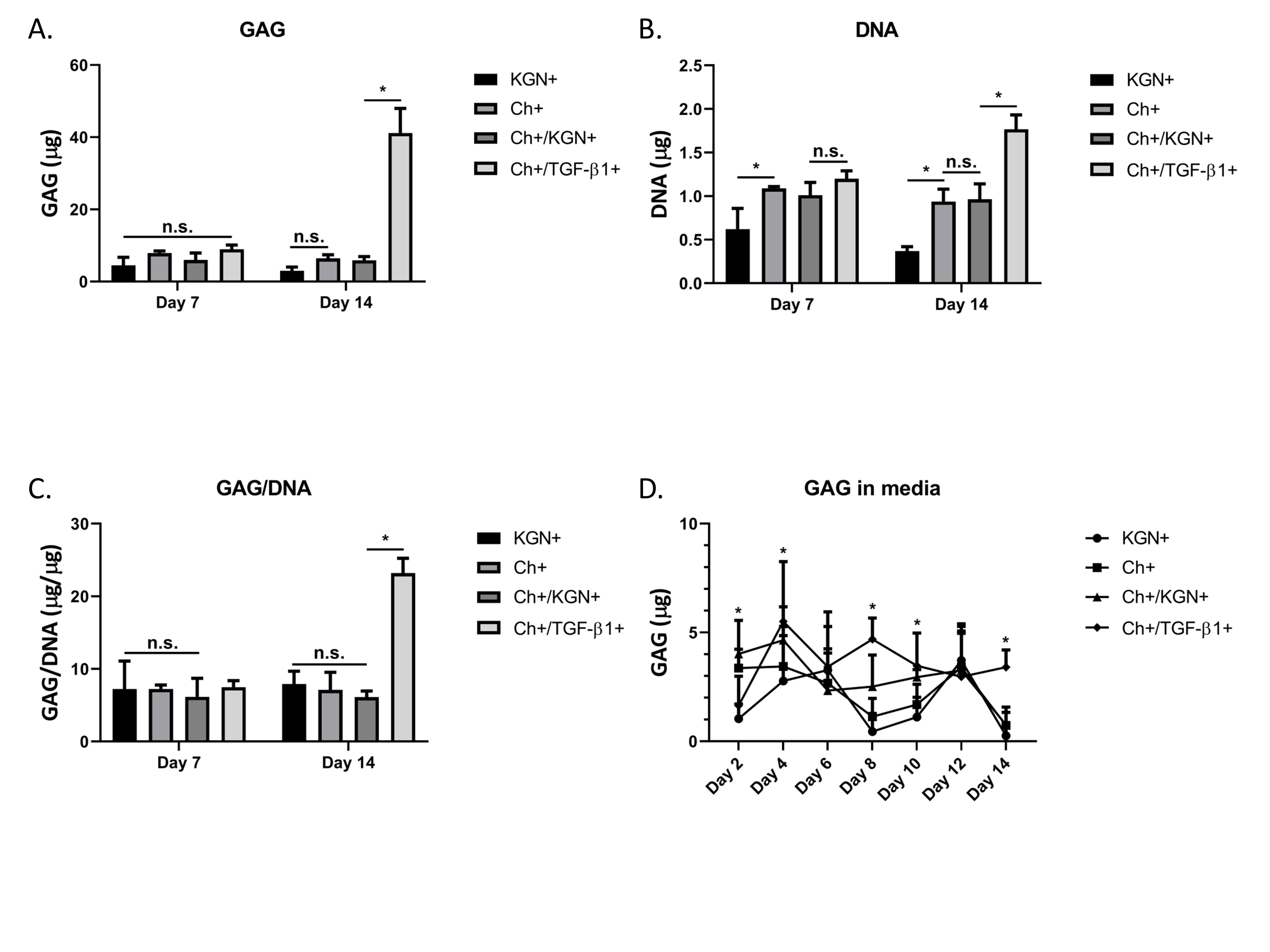

Supplement: Supplementary file 10 — Supplementary Figure 10. [file 41598_2020_65283_MOESM10_ESM.tif]

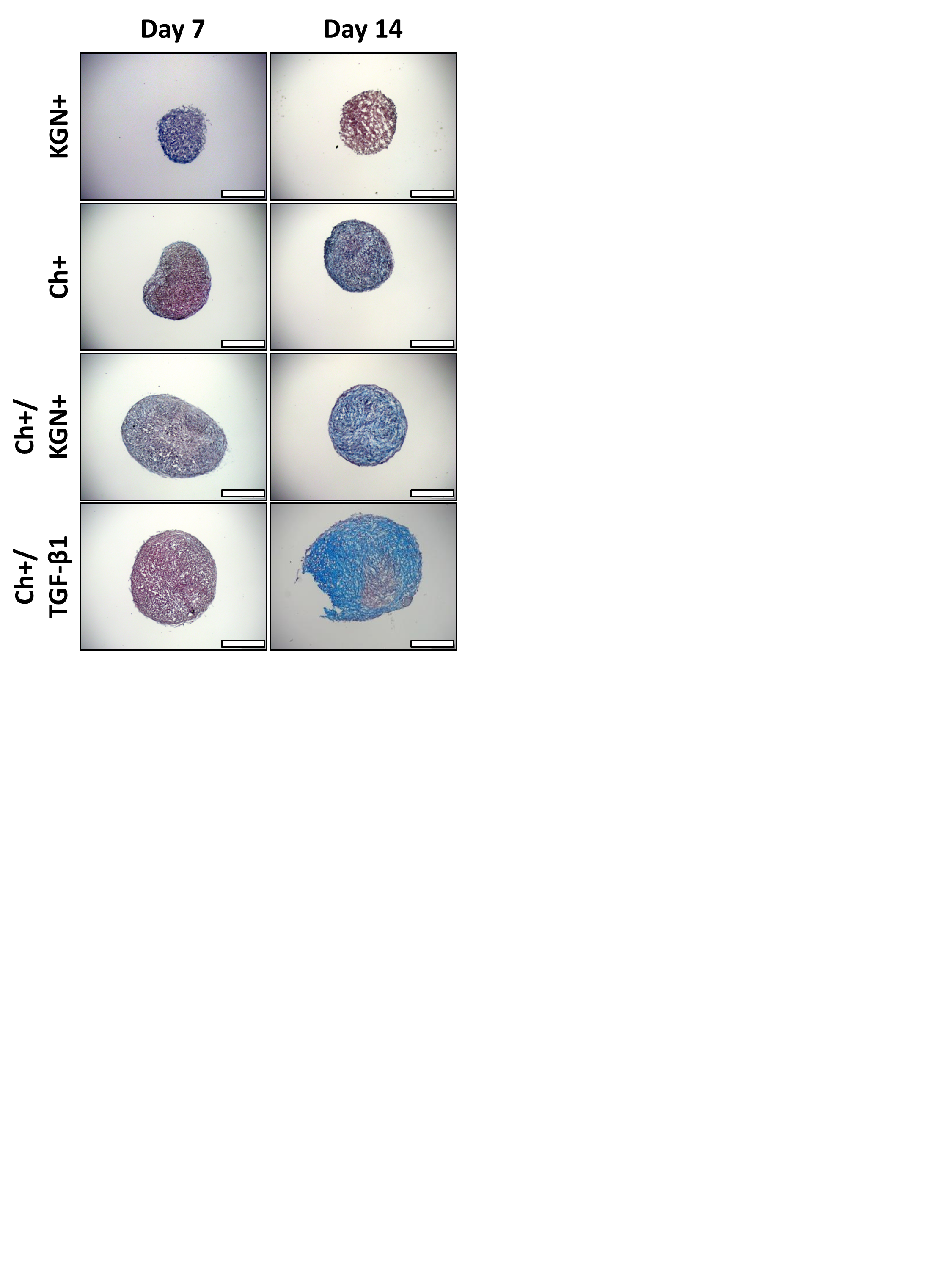

Supplement: Supplementary file 11 — Supplementary Figure 11. [file 41598_2020_65283_MOESM11_ESM.tif]
