# Supplementary material for: Transforming growth factor-beta stimulates human bone marrow-derived mesenchymal stem/stromal cell chondrogenesis more so than kartogenin
Source: Sci Rep. 2020 May 20;10:8340. doi: 10.1038/s41598-020-65283-8 (PMC7239921; doi:10.1038/s41598-020-65283-8)
Supplement: Supplementary file 12 — Supplementary Figures and Legends. [file 41598_2020_65283_MOESM12_ESM.docx]

**Supplementary Data File**

**Transforming growth factor-beta stimulates human bone marrow-derived mesenchymal stem/stromal cell chondrogenesis more so than kartogenin**

**Music, E.^1,2^, Klein, T.J.^3^, Lott, W.B. ^1,2,3^, Doran, M.R.^1,2,3-7***^**

*^1^ Doran Laboratory, School of Biomedical Sciences, Faculty of Health, Institute of Health and Biomedical Innovation (IHBI), Queensland University of Technology (QUT), Brisbane, Australia*

*^2^ Translational Research Institute (TRI), Brisbane, Australia*

*^3^ School of Chemistry, Physics and Mechanical Engineering (CPME), Science and Engineering Faculty (SEF), Institute of Health and Biomedical Innovation (IHBI), Queensland University of Technology (QUT), Brisbane, Australia*

*^4^ Australian Prostate Cancer Research Centre – Queensland (APCRC-Q), Brisbane, Australia*

*^5^ Mater Research Institute – University of Queensland (UQ), Translational Research Institute (TRI), Brisbane, Australia*

*^6^ National Centre for the Public Awareness of Science, Australian National University (ANU), Canberra, Australia*

*^7^ Craniofacial and Skeletal Diseases Branch, National Institute of Dental and Craniofacial Research (NIDCR), National Institutes of Health (NIH), Department of Health and Human Services, Bethesda, Maryland, USA*

***Correspondence to: [michael.doran@qut.edu.au](mailto:michael.doran@qut.edu.au)

**Supplementary Data**


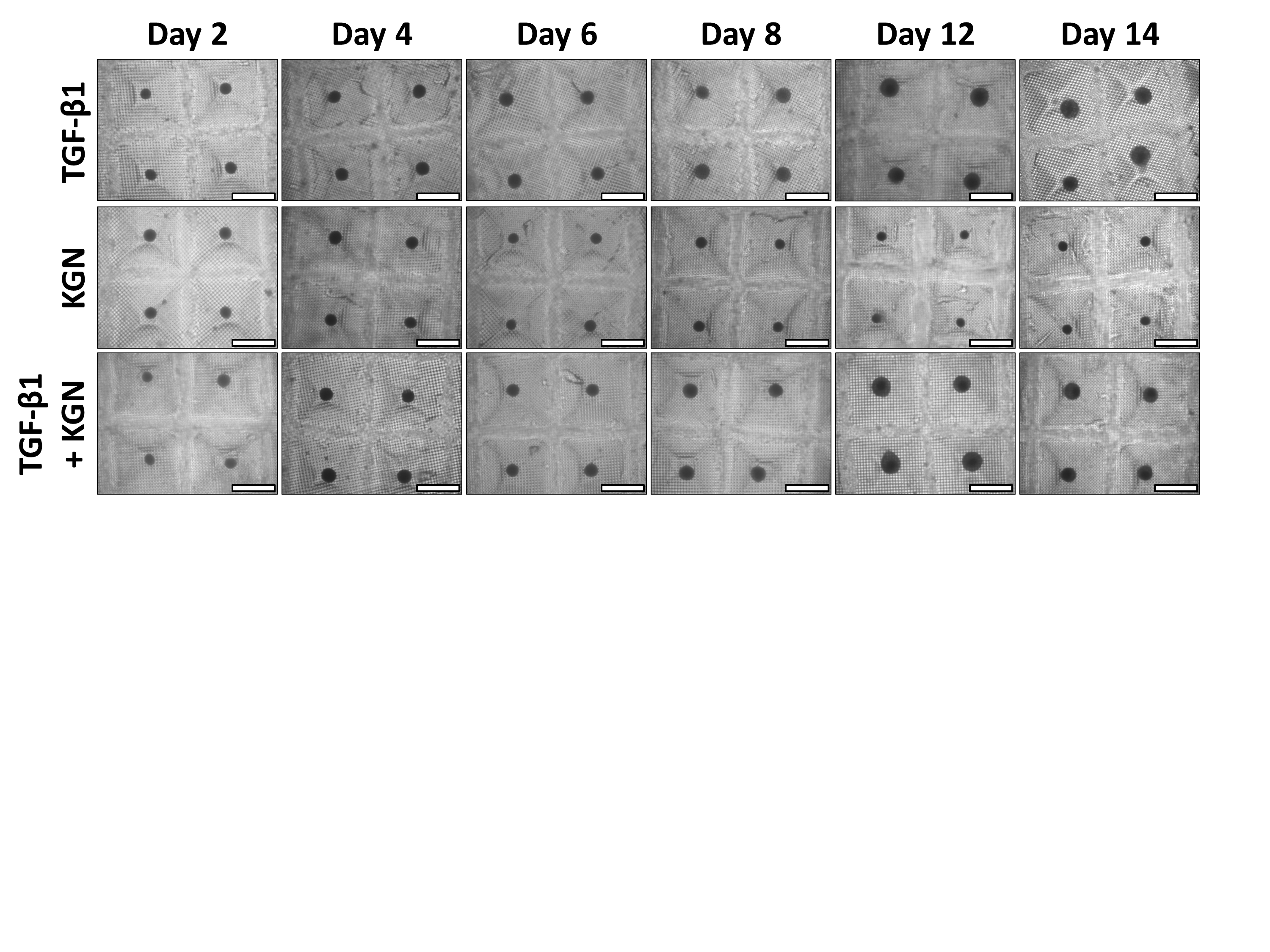


**Supplementary Figure 1.** BMSC Donor 2. Microscope images of micropellets over a 14-day culture period. Micropellets were imaged throughout the differentiation period. At Day 14, micropellets in the two groups containing TGF-β1 were larger than those in the KGN alone group. Scale bar = 1 mm.

**
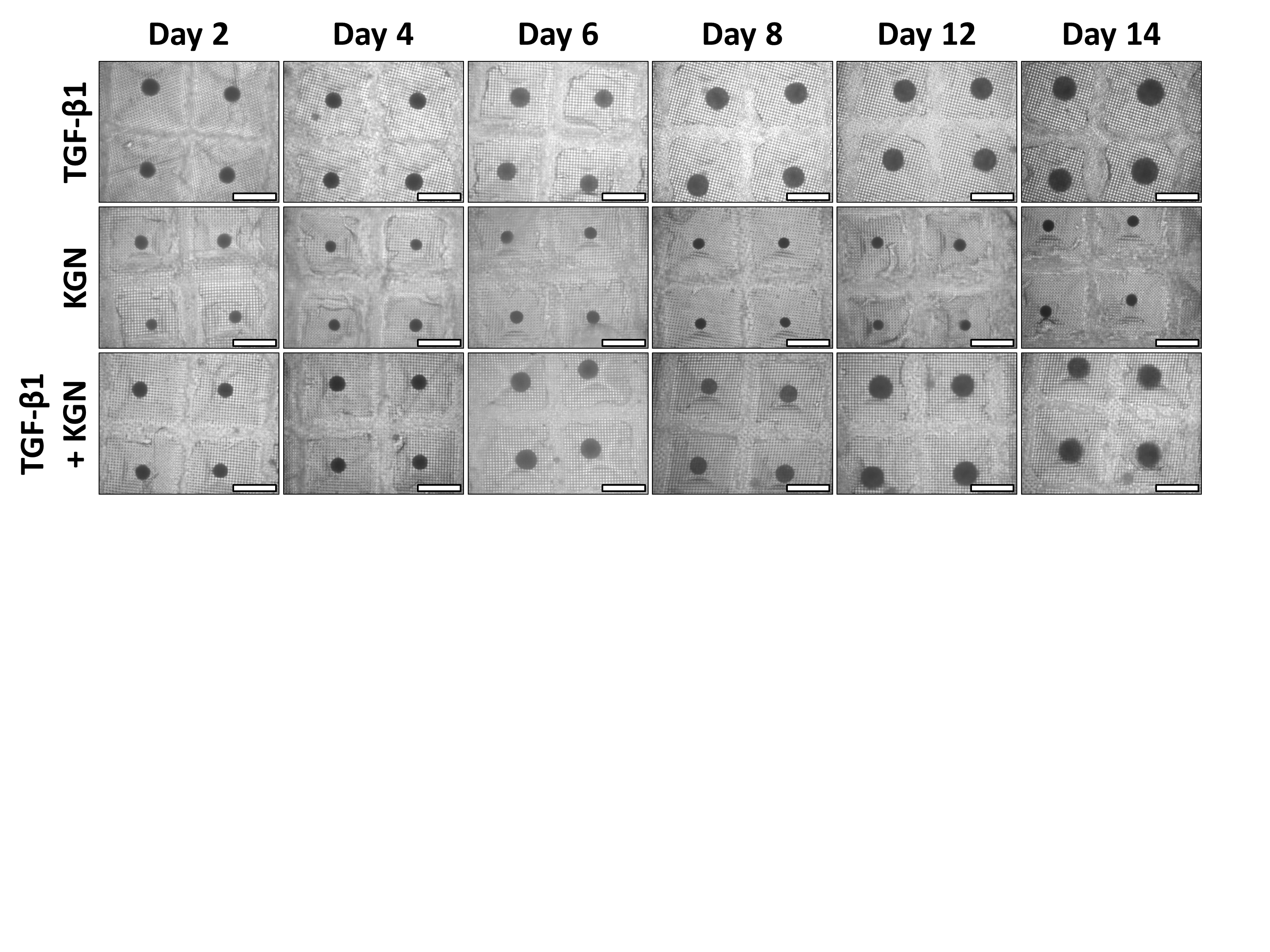
**

**Supplementary Figure 2.** BMSC Donor 3. Microscope images of micropellets over a 14-day culture period. Micropellets were imaged throughout the differentiation period. At Day 14, micropellets in the two groups containing TGF-β1 were larger than those in the KGN alone group. Scale bar = 1 mm.


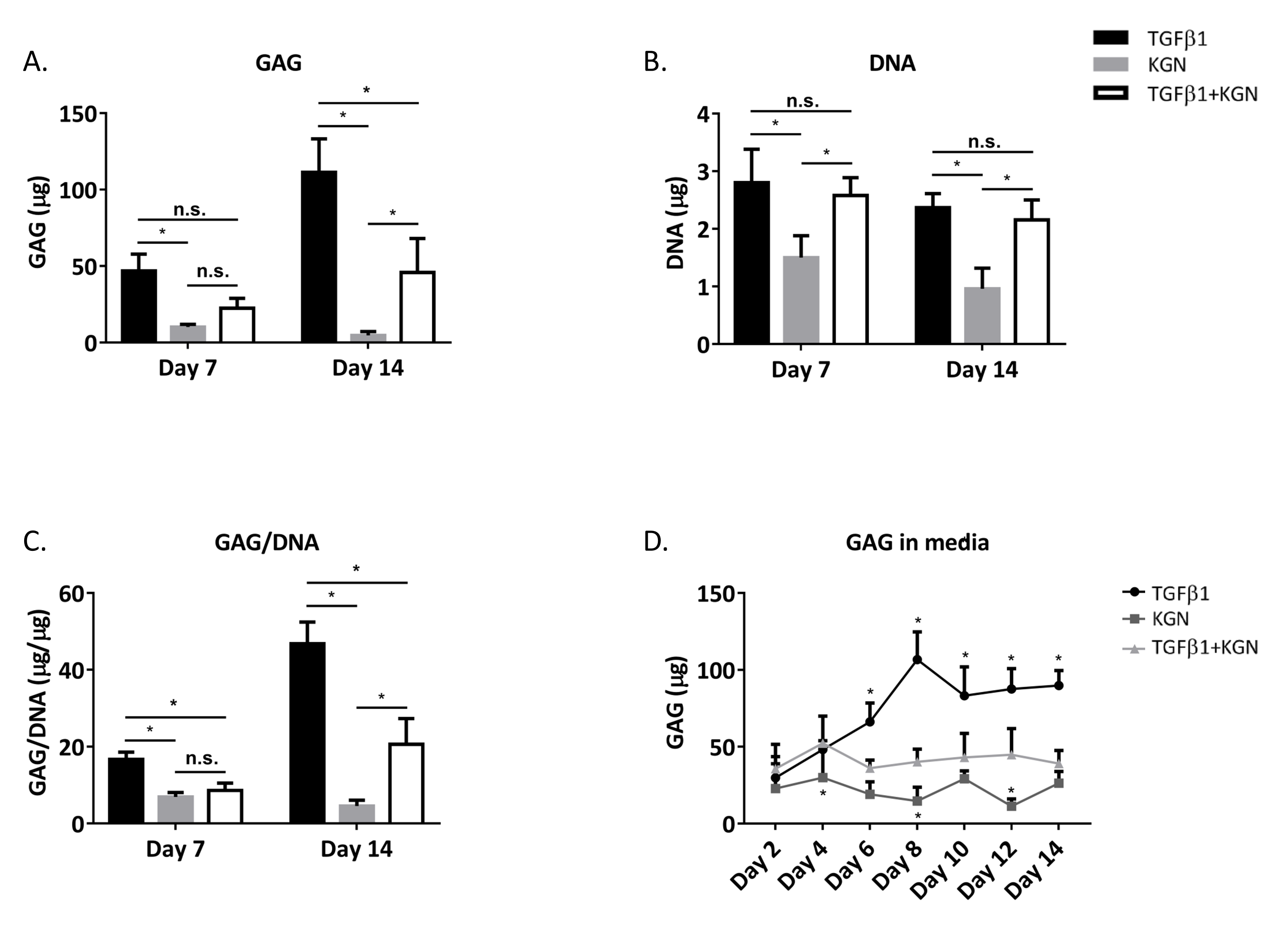


**Supplementary Figure 3.** BMSC Donor 2. GAG and DNA quantities in micropellets, GAG/DNA, and GAG secreted to media. A) Quantities of GAG in micropellets at Day 7 and Day 14. B) DNA quantities in micropellets. C) GAG normalized to DNA in micropellets. D) Quantification of GAG secreted to the media by micropellets over a 14-day culture period. For A-C, mean ± SD, n = 4, P < 0.05; for D, mean ± SD, n = 6, P < 0.05.

**
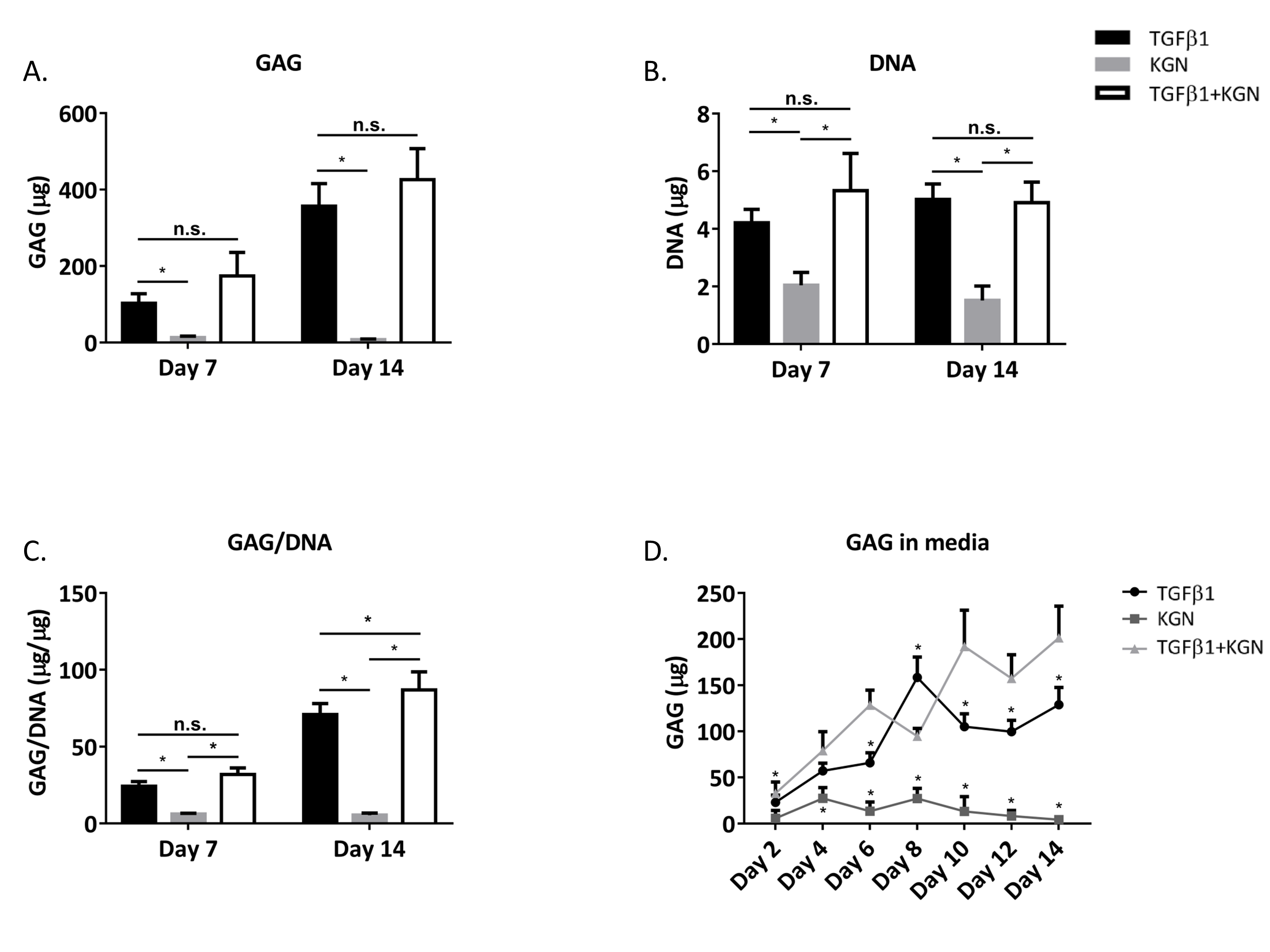
**

**Supplementary Figure 4.** BMSC Donor 3. GAG and DNA quantities in micropellets, GAG/DNA, and GAG secreted to media. A) Quantities of GAG in micropellets at Day 7 and Day 14. B) DNA quantities in micropellets. C) GAG normalized to DNA in micropellets. D) Quantification of GAG secreted to the media by micropellets over a 14-day culture period. For A-C, mean ± SD, n = 4, P < 0.05; for D, mean ± SD, n = 6, P < 0.05.

**
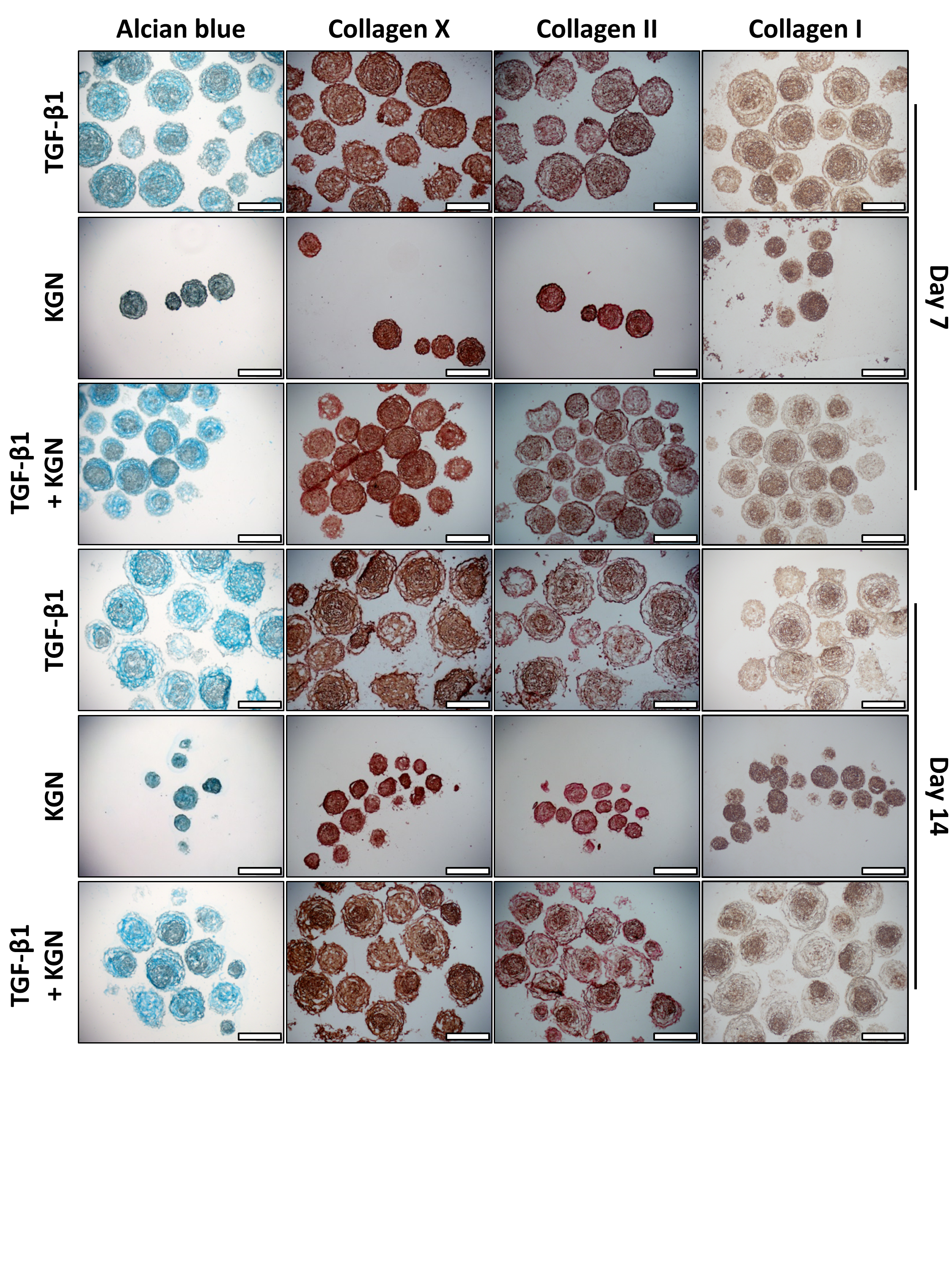
**

**Supplementary Figure 5.** BMSC Donor 2. Histology: Alcian blue and type X, II and I collagen. Alcian blue (first column) was used to stain sections of micropellets. Type X collagen immunohistochemical staining of sections of micropellets is shown in the second column. The third column shows type II collagen immunohistochemical staining of sections of micropellets. The final column shows type I collagen immunohistochemical staining of sections of micropellets. Scale bar = 400 μm.


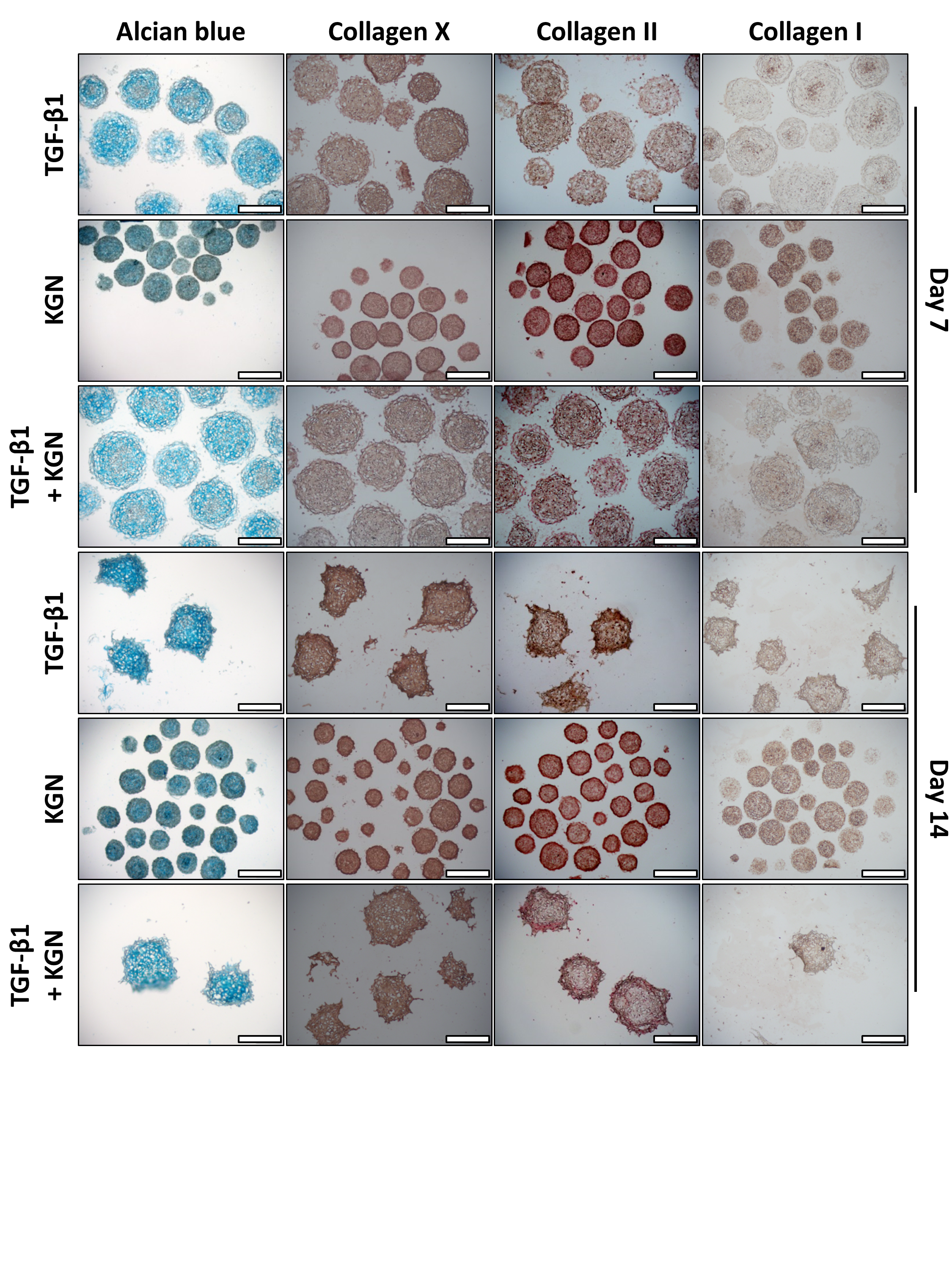


**Supplementary Figure 6.** BMSC Donor 3. Histology: Alcian blue and type X, II and I collagen. Alcian blue (first column) was used to stain sections of micropellets. Type X collagen immunohistochemical staining of sections of micropellets is shown in the second column. The third column shows type II collagen immunohistochemical staining of sections of micropellets. The final column shows type I collagen immunohistochemical staining of sections of micropellets. Scale bar = 400 μm.


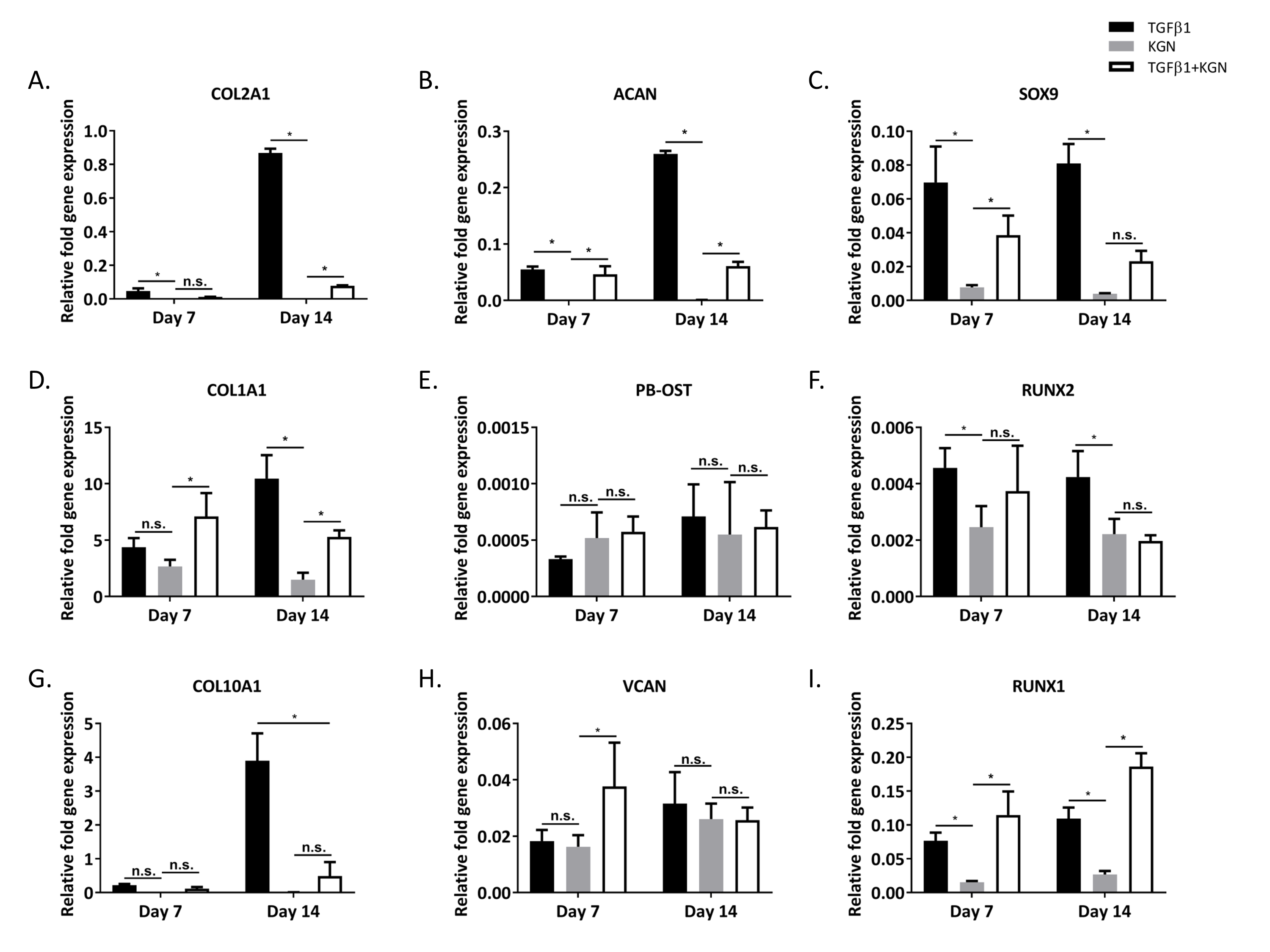


**Supplementary Figure 7.** BMSC Donor 2. qRT-PCR analysis of cartilage-like tissues generated from BMSC. Increased expression of chondrogenic markers was observed in both TGF-β1 conditions compared to KGN alone. Expression levels are shown relative to RPLPO gene expression (plotted as mean ± SD, n = 4, P < 0.05).


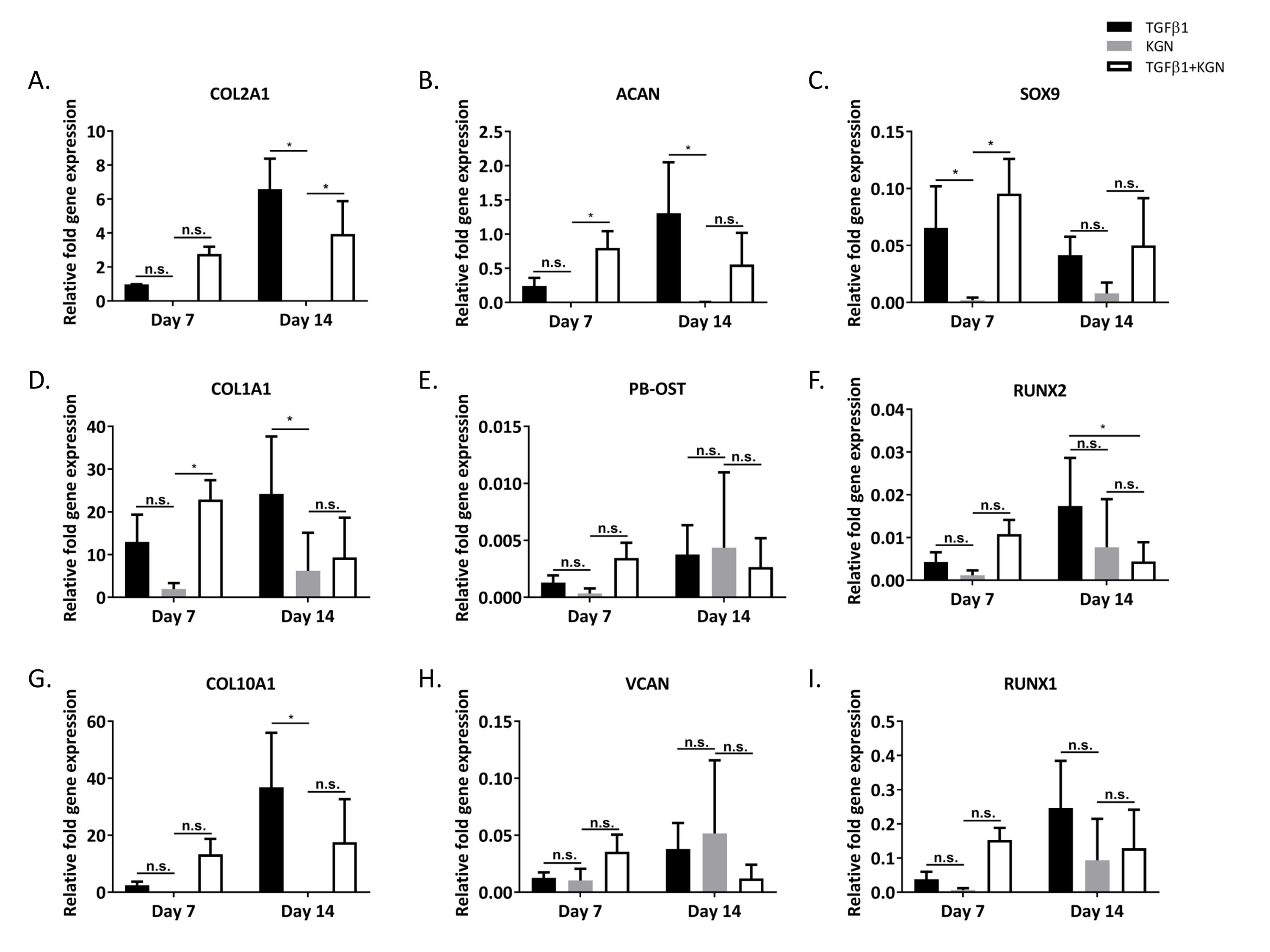


**Supplementary Figure 8.** BMSC Donor 3. qRT-PCR analysis of cartilage-like tissues generated from BMSC. Increased expression of chondrogenic markers was observed in both TGF-β1 conditions compared to KGN alone. Expression levels are shown relative to RPLPO gene expression (plotted as mean ± SD, n = 4, P < 0.05).

**
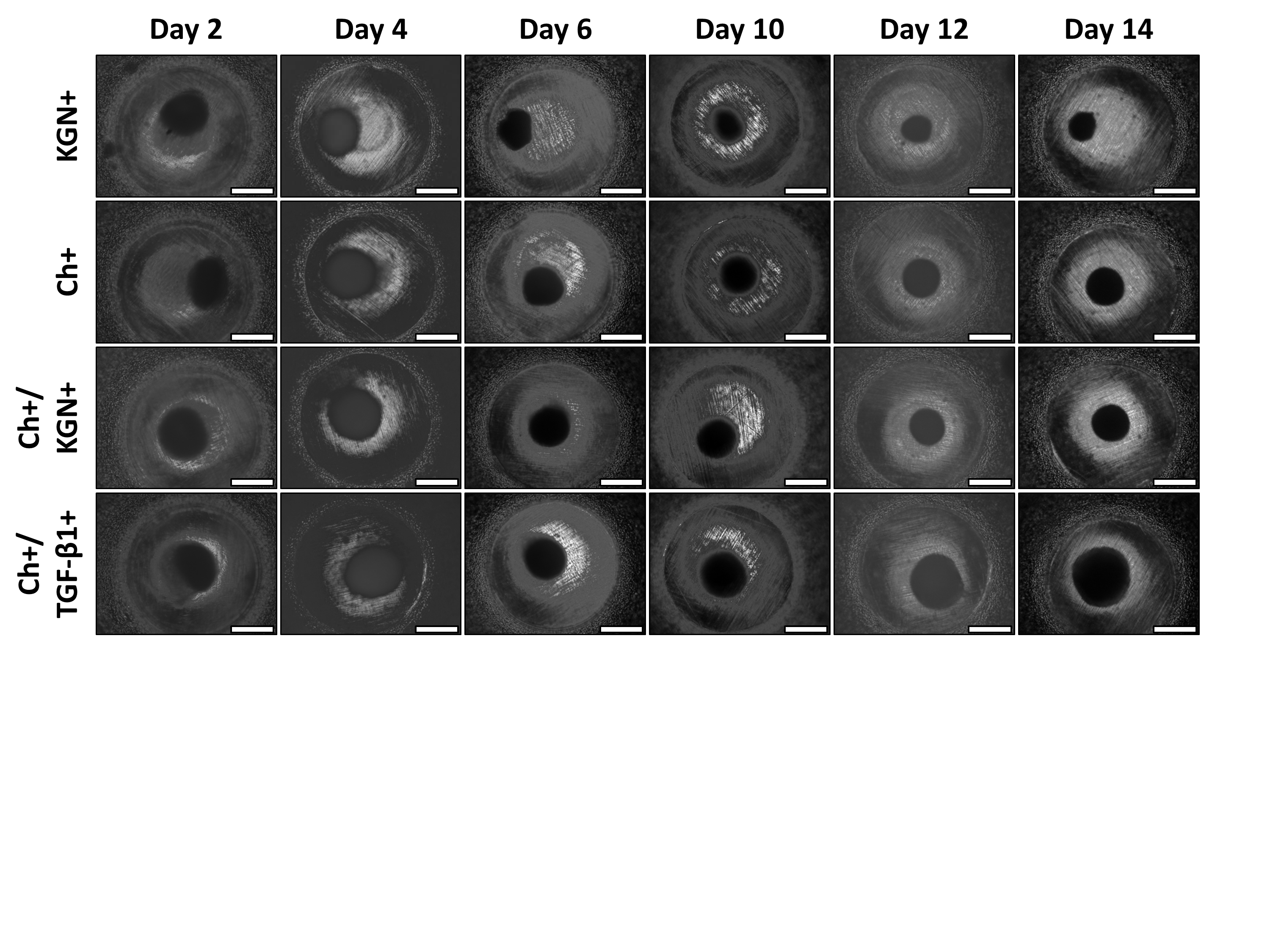
**

**Supplementary Figure 9.** Microscope images of macropellets over a 14-day culture period. Macropellets were imaged throughout the differentiation period. At Day 14, macropellets in the group containing TGF-β1 were larger in diameter than those in the other three groups, including the two KGN groups. Medium conditions were (1) kartogenin (KGN), (2) Chondrogenic medium (Ch+), (3) Chondrogenic medium + kartogenin (Ch+/KGN+), and (4) Chondrogenic medium + TGF-β1 (Ch+/ TGF-β1+). Scale bar = 1 mm.

**
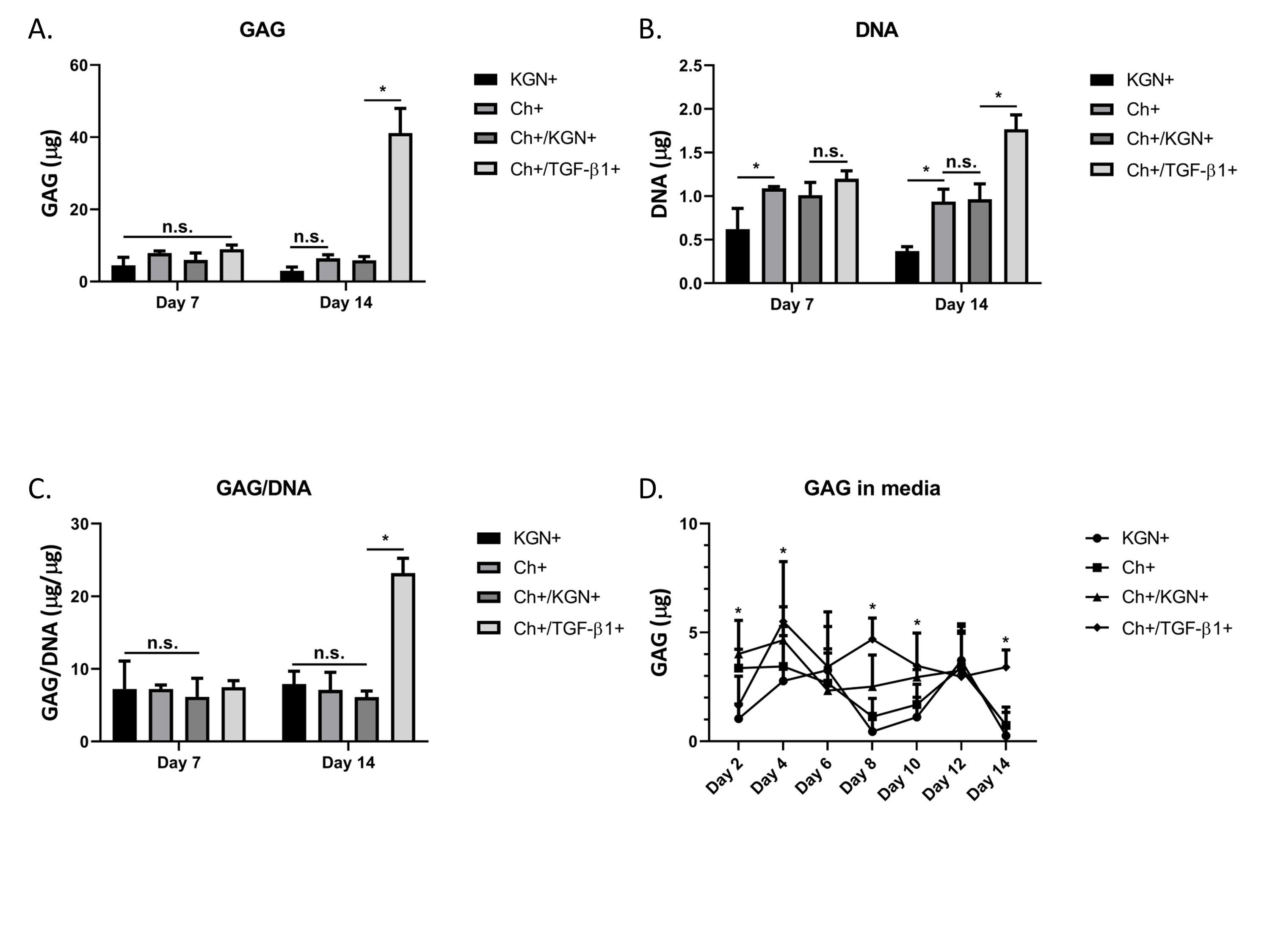
**

**Supplementary Figure 10.** GAG and DNA quantities in macropellets, GAG/DNA, and GAG secreted to media. A) Quantities of GAG in macropellets at Day 7 and Day 14. B) DNA quantities in macropellets. C) GAG normalized to DNA in macropellets. D) Quantification of GAG secreted to the media by macropellets over a 14-day culture period. Medium conditions were (1) Kartogenin (KGN), (2) Chondrogenic medium (Ch+), (3) Chondrogenic medium + kartogenin (Ch+/KGN+), and (4) Chondrogenic medium + TGF-β1 (Ch+/ TGF-β1+). Mean ± SD, n = 4, P < 0.05.


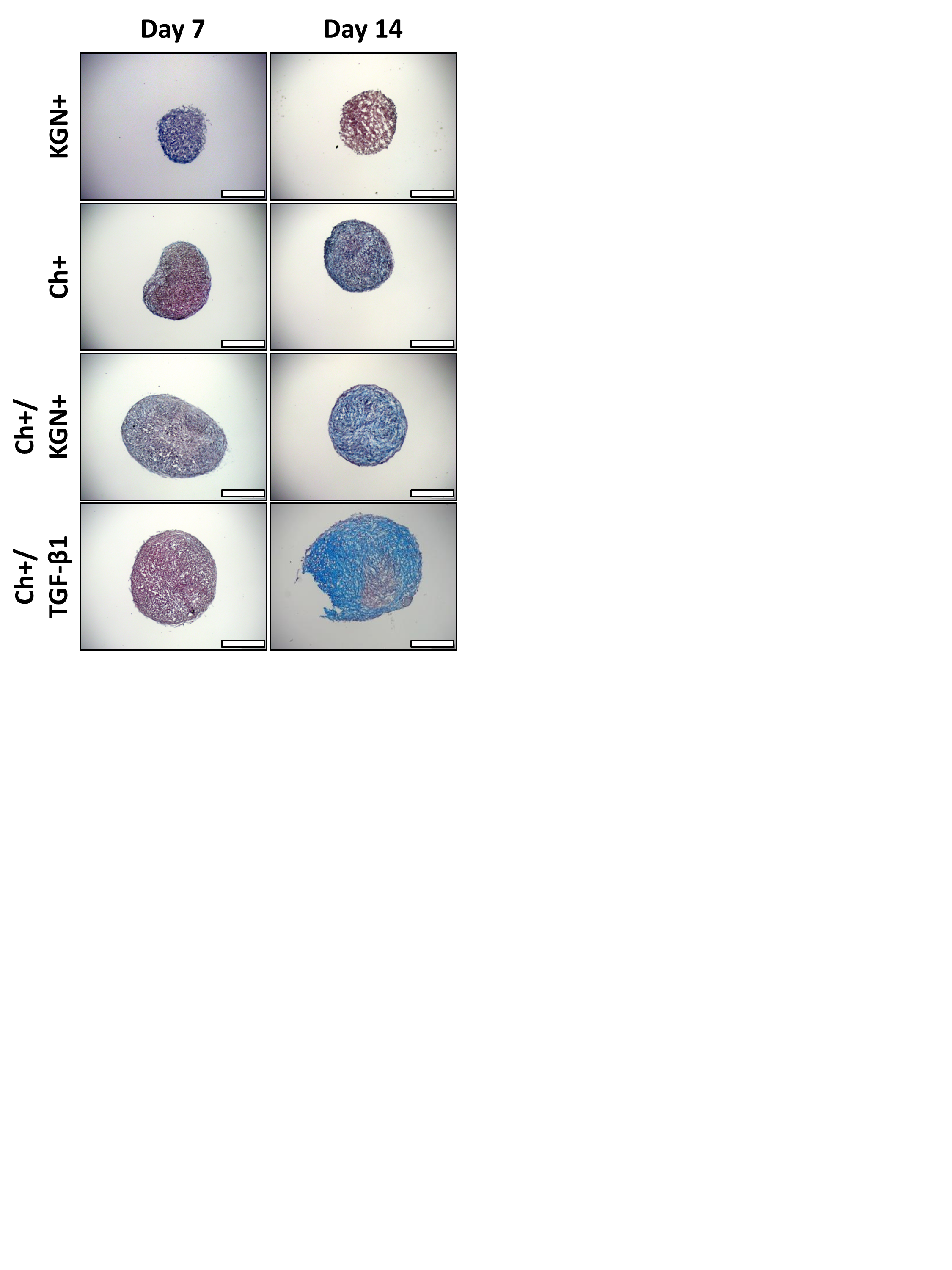


**Supplementary Figure 11.** Alcian blue staining of macropellets. Alcian blue was used to stain sections of Day 7 and Day 14 macropellets. Medium conditions were (1) Kartogenin (KGN), (2) Chondrogenic medium (Ch+), (3) Chondrogenic medium + kartogenin (Ch+/KGN+), and (4) Chondrogenic medium + TGF-β1 (Ch+/ TGF-β1+). Scale bar = 400 μm.
